# Global exploration of the metabolic requirements of gallid alphaherpesvirus 1

**DOI:** 10.1371/journal.ppat.1008815

**Published:** 2020-08-24

**Authors:** Yangyang Qiao, Zhitao Wang, Zongxi Han, Yuhao Shao, Yong Ma, Yumeng Liang, Zhijie Chen, Hanguang Wu, Lu Cui, Yanhui Zhang, Shengwang Liu, Hai Li

**Affiliations:** 1 Division of Avian Infectious Diseases, State Key Laboratory of Veterinary Biotechnology, Harbin Veterinary Research Institute, the Chinese Academy of Agricultural Sciences, Harbin, the People’s Republic of China; 2 College of Veterinary Medicine, Northeast Agricultural University, Harbin, the People’s Republic of China; University of Southern California, UNITED STATES

## Abstract

Although therapeutics targeting viral metabolic processes have been considered as promising strategies to treat herpesvirus infection, the metabolic requirements of gallid alphaherpesvirus 1 (ILTV), which is economically important to the poultry industry worldwide, remain largely unknown. Using the ILTV-susceptible but nonpermissive chicken cell line DF-1 and the ILTV-permissive chicken cell line LMH as models, the present study explored the metabolic requirements of ILTV by global transcriptome analysis and metabolome assays of ILTV infected cell lines in combination with a set of functional validations. The extensive metabolic exploration demonstrated that ILTV infection tended to promote a metabolic shift from glycolysis to fatty acid (FA) and nucleotide biosynthesis and utilizes glutamine independently of glutaminolysis, without significant general effect on the TCA cycle. In addition, different metabolic pathways were found to be required for distinct stages of ILTV replication. Glucose and glutamine were required for the transcription of viral immediate early gene *ICP4* and subsequent steps of viral replication. However, FA synthesis was essential for assembly but not required for other upstream steps of ILTV replication. Moreover, the metabolic requirements of ILTV infection revealed in chicken cell lines were further validated in chicken primary cells isolated from chicken embryo kidneys and chicken embryo livers. The present study, to the best of our knowledge, provides the first global metabolic profile of animal herpesviruses and illustrates the main characteristics of the metabolic program of ILTV.

## Author summary

Virus-host metabolic interaction is a promising target for antiviral therapeutics. Explorations of viral-induced shifts of host metabolism could reveal substrates that are uniquely required at high levels for viral replication and have been conducted for many virus species in the last decade. Herpesviruses are commonly treated with nucleotide analogs in the clinic. However, recent metabolomics studies suggest that different herpesvirus species can execute distinct metabolic programs in host cells despite their high conservation of biological characteristics. To understand why herpesviruses have evolved to alter different metabolic processes in host cells and refine the antiviral treatments by targeting more species-specific metabolic requirements, metabolomic explorations of more herpesvirus species are needed. However, comprehensive exploration of virus-host metabolic interaction has only been limited to a few human herpesviruses. Gallid alphaherpesvirus 1 (ILTV), an economically important alphaherpesvirus to the poultry industry worldwide, is thought to be an ancient example of alphaherpesviruses. Here we reveal the global metabolic requirements of ILTV and highlight the common and unique metabolic characteristics of ILTV by comparing with the known global metabolic profiles of several human herpesviruses. Our study may increase current understanding of herpesvirus-host metabolic interplay and inspire further studies in this direction.

## Introduction

The host cell metabolism provides viruses with all energy and macromolecules required for viral replication and thereby is a promising target for antiviral therapeutics. Recently, significant efforts have been made in the examination of the host cell metabolism during virus infection to reveal the metabolic requirements of virus replication. Some antiviral drugs targeting the metabolic processes essential for the viral life cycle have been proven to be effective in the clinic in the treatments of many human RNA and DNA viruses, including hepatitis B virus (HBV), hepatitis C virus (HCV), human immunodeficiency virus (HIV), human cytomegalovirus (HCMV), varicella-zoster virus (VZV), and herpes simplex virus (HSV) [1]. Accumulating evidence suggests that even viruses from the same family can execute distinct metabolic programs in host cells. For example, HCMV increases the host glycolytic flux to feed the tricarboxylic acid (TCA) cycle for ultimate fatty acid (FA) synthesis; in contrast, HSV-1 mainly increases the anaplerotic influx to the TCA cycle through pyruvate carboxylase to feed pyrimidine biosynthesis [2]. Although both viruses are currently treated with drugs targeting nucleotide metabolism in the clinic, therapeu tic interventions at other points in the metabolic flux from glycolysis to fatty acid synthesis might prove more effective for the treatment of HCMV. However, the investigation of virus-host metabolic interaction is still at its very early stage, and the metabolic requirements of most viruses remain unclear. Therefore, extensive investigations of the metabolic requirements of more viruses are urgently needed to refine the rational design of virus-specific therapeutics.

*Herpesviridae* have three known subfamilies that include *Alphaherpesvirinae*, *Betaherpesvirinae*, and *Gammaherpesvirinae* and have a broad host range from humans to fish [3]. However, comprehensive exploration of virus-host metabolic interactions has only been applied for human herpesviruses, including HCMV, VZV, HSV, Kaposi sarcoma-associated herpesvirus (KSHV), and Epstein-Barr virus (EBV) [2, 4–8]. Gallid alphaherpesvirus 1 (GaHV-1), also known as avian infectious laryngotracheitis virus (ILTV), which belongs to the family *Herpesviridae* and the subfamily *Alphaherpesvirinae*, continues to cause substantial economic losses to the poultry industry worldwide [9]. Similar to human alphaherpesviruses, due to the establishment of latent infection in the trigeminal ganglia after acute infection of the upper respiratory tract, ILTV cannot be cleared from the host and no effective therapeutic treatment is available currently. Previous *in vitro* and *in vivo* studies, including ours, have shown that ILTV infection blocks apoptosis of infected cells, thereby prolonging the lifespan of infected cells and consequently facilitating viral replication [10–12]. During this process, many metabolic signaling pathways are altered in host cells, which might play an important role in ILTV replication. At minimum, fatty acid metabolism has been found to be essential for the cell-to-cell transmission of ILTV [13]. Therefore, elucidation of the metabolic requirements of ILTV infection may increase current understanding of herpesvirus-host metabolic interplay and inspire further studies in this direction. However, the metabolic requirements of ILTV remain largely unclear.

One limitation of the metabolic exploration of ILTV infection is the selection of a proper experimental model. ILTV has a limited host range both *in vivo* and *in vitro*. *In vitro*, ILTV can be efficiently propagated only in kidney and liver cell cultures primarily isolated from chickens or chicken embryos, including chicken kidney cells (CK), chicken embryo kidney cells (CEK), chicken embryo liver cells (CEL), and the chicken liver hepatocellular carcinoma cell line LMH, but not in cells isolated from other tissues or any other available chicken cell lines [14]. Although primary cells can better mimic the *in vivo* process of virus infection, the complexity, heterogeneity, and short lifespan of primary cells during *in vitro* culture limit their use in mechanistic studies. Thus, LMH cells have been commonly used for the investigation of ILTV infection and proven to be a reliable *in vitro* model [12, 13, 15–18]. However, despite being capable of exploring the metabolic characteristics of ILTV, this model may not be sensitive enough to investigate metabolic shift at the early stage of virus infection, such as the initial stage of viral transcription, due to that tumor cells often have distinct metabolic patterns to meet the rapid energy requirements [19, 20]. DF-1 is another widely used immortalized cell line derived from chicken embryo fibroblasts (CEFs) and has been shown to be nonpermissive to ILTV replication [14]. In the present study, we found that, despite being nonpermissive, DF-1 cells are susceptible to ILTV allowing transient transcription of early viral genes. Further transcriptome analysis revealed the shifts of cellular metabolic pathways as the main difference between DF-1 cells and LMH cells upon ILTV infection. Therefore, DF-1 cells and LMH cells infected with ILTV were used as models to explore the metabolic processes essential for ILTV transcription and the following viral replication steps. Metabolism is broadly defined as the sum of biochemical processes in living organisms. To achieve comprehensive analysis of the entire metabolome, liquid chromatography coupled to mass spectrometry (LC-MS) was employed in the present study. ILTV-host cell metabolic interactions were extensively explored by metabolome analysis in combination with functional validations.

## Results

### DF-1 cells and LMH cells have similar susceptibility but distinct permissibility to ILTV

**Fig 1:**
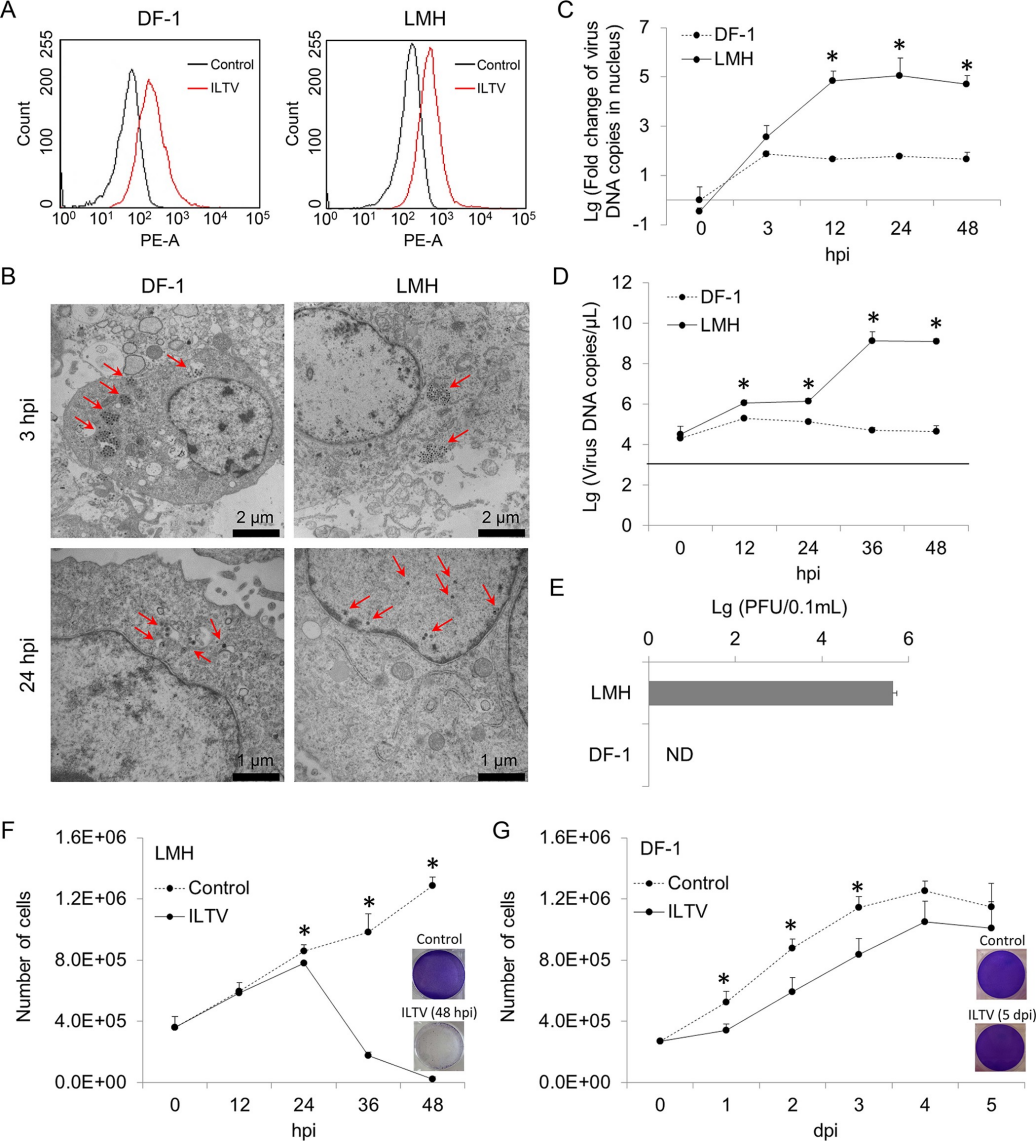
Biological characterization of ILTV infection in LMH cells and DF-1 cells. (A) Flow cytometry diagram of the membrane fusion showing in LMH and DF-1 cells infected with ILTV (MOI 1) at 3 hours post infection (hpi). (B) Transmission electron microscopy images of ILTV-infected LMH and DF-1 cells (MOI 5) at 3 and 24 hpi. Representative images are shown for each condition. The arrows show viral particle locations within the cell. (C) The numbers of viral genome copies in cell nuclei of DF-1 and LMH cells were determined by ILTV-specific qPCR (MOI 0.1). (D and E) The replication of ILTV in DF-1 and LMH cells was determined by ILTV specific absolute RT-qPCR (D) and plaque assays (E), respectively (MOI 0.1). The dotted line in D indicates detect limit. (F and G) The growth curves of LMH (F) and DF-1 (G) cells after ILTV infection (MOI 1). The number of adherent cells that were negative for trypan blue staining was counted. The cytopathic effect of ILTV infection on cells was visualized by crystal violet staining. The results in C to G are presented as the mean ± SD, n = 3. Asterisks indicate statistical difference (*p* < 0.05).

**Fig 2:**
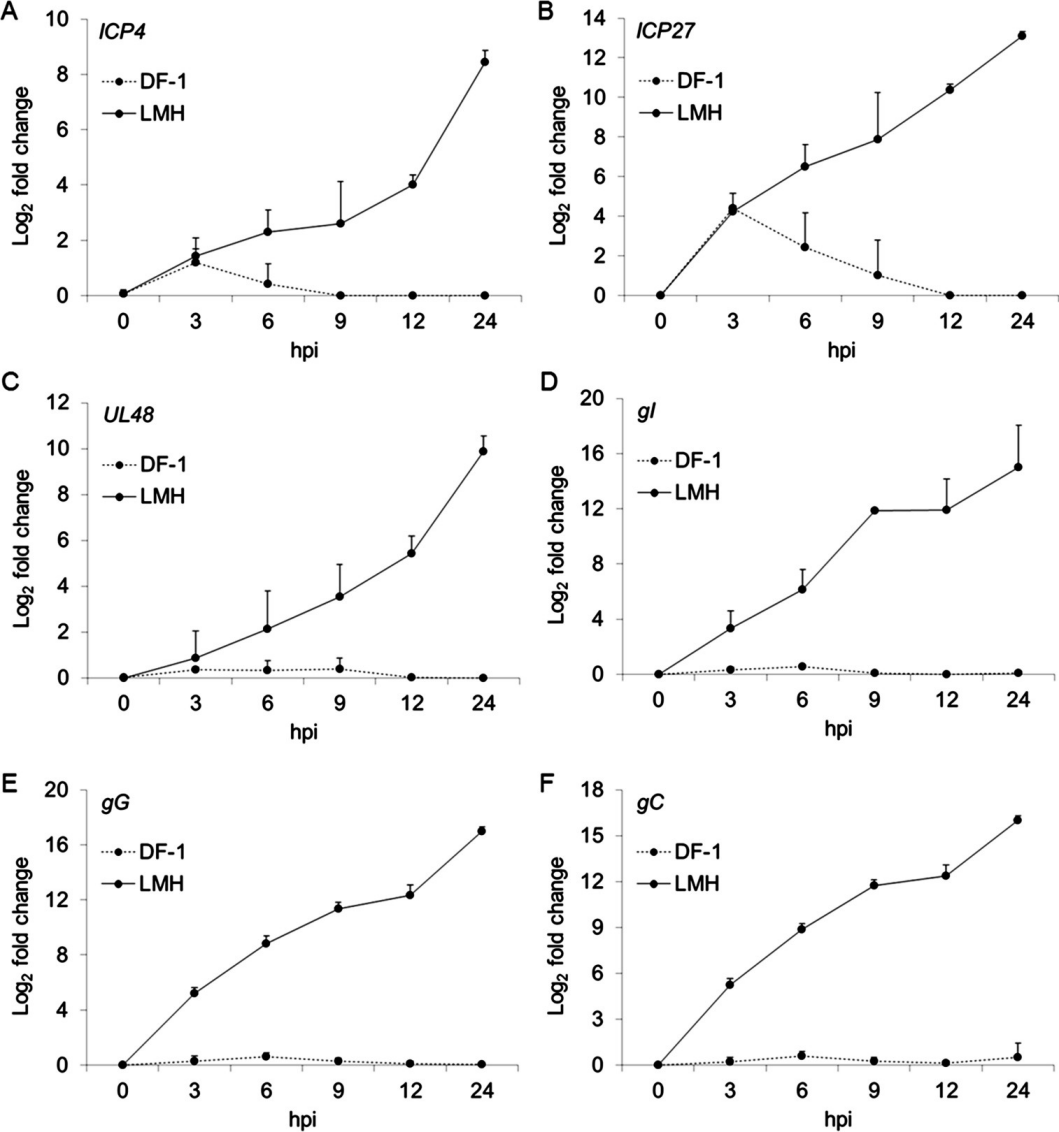
Detection of viral gene transcription in LMH cells and DF-1 cells. The transcription levels of six ILTV genes coving all stages of ILTV transcription, namely *ICP4* (A), *ICP27* (B), *UL48* (C), *gI* (D), *gG* (E), and *gC* (F), in DF-1 and LMH cells were detected by absolute RT-qPCR at indicated time points post ILTV infection (MOI 1). Data are presented as the mean ± SD, n = 3.

To study the susceptibility of DF-1 cells and LMH cells to ILTV, the fusion between the viral membrane and host cell membrane was detected by FACS using virus preincubated with octadecyl rhodamine B chloride (R18), a lipophilic dye widely used for virus penetration detection. Clear diffusion of R18 was observed on the membranes of both DF-1 cells and LMH cells, suggesting that the ILTV membrane could fuse with the membranes of both cell lines (Fig 1A). The entry of ILTV into these two cell lines was further evidenced by electron microscopic observation of viral particles in the cells (Fig 1B). These results suggest that both DF-1 cells and LMH cells are susceptible to ILTV. For herpesviruses, both the replication of the genome and the packaging of viral particles are completed in the host cell nucleus. Interestingly, electron microscopic observation only found viral particles in the nuclei of LMH cells but not in the nuclei of DF-1 cells (Fig 1B), indicating differences in either the entry of viral genomes into the nucleus or the packaging of viral particles between these two cell lines. To address this issue, the presence of viral genomes in host cell nuclei and the production of virions were detected. Similar amounts of viral genomes were observed in the nuclei of DF-1 cells and LMH cells at 3 hours post infection (hpi) as assayed by ILTV-specific absolute quantitative real-time PCR (qPCR), while an increase in the viral genome copy number with increasing time of infection was only observed in the nuclei of LMH cells, which reached a plateau at 12 hpi (Fig 1C), suggesting that viral genomes could be delivered into the nuclei of DF-1 cells but failed to replicate. Consistent results were observed in the detection of the total amount of viral genomes with a longer duration of viral genome replication before reaching a plateau at 36 hpi (Fig 1D). As a consequence, infectious virions were only observed in LMH cells but not in DF-1 cells as demonstrated by plaque assay (Fig 1E), suggesting that LMH cells but not DF-1 cells are permissive to ILTV. In addition, the cytopathogenic effect (CPE) of ILTV was only observed in LMH cells together with a drop in cell numbers at 24 hpi (Fig 1F). Despite no CPE observed, transient growth suppression was induced by ILTV in DF-1 cells (Fig 1G). To gain further insight into the difference in the genome replication of ILTV between DF-1 cells and LMH cells, viral transcription of six ILTV genes covering all stages of ILTV transcription were detected by RT-qPCR (Fig 2). The transcription levels of all these genes constantly increased during the time of detection in LMH cells. In contrast, only the transcription of immediate early gene (IEG) *ICP4* and early gene (EG) *ICP27* were observed within 9 hpi in DF-1 cells with peak expression at 3 hpi.

Taken together, the above findings demonstrate that DF-1 cells are susceptible but not permissive to ILTV infection because DF-1 cells only support transient transcription of IEG and EG of ILTV. Thus, DF-1 cells and LMH cells can be used as *in vitro* model to investigate the requirements for ILTV transcription.

### Transcriptome analysis of ILTV-infected cells

**Fig 3:**
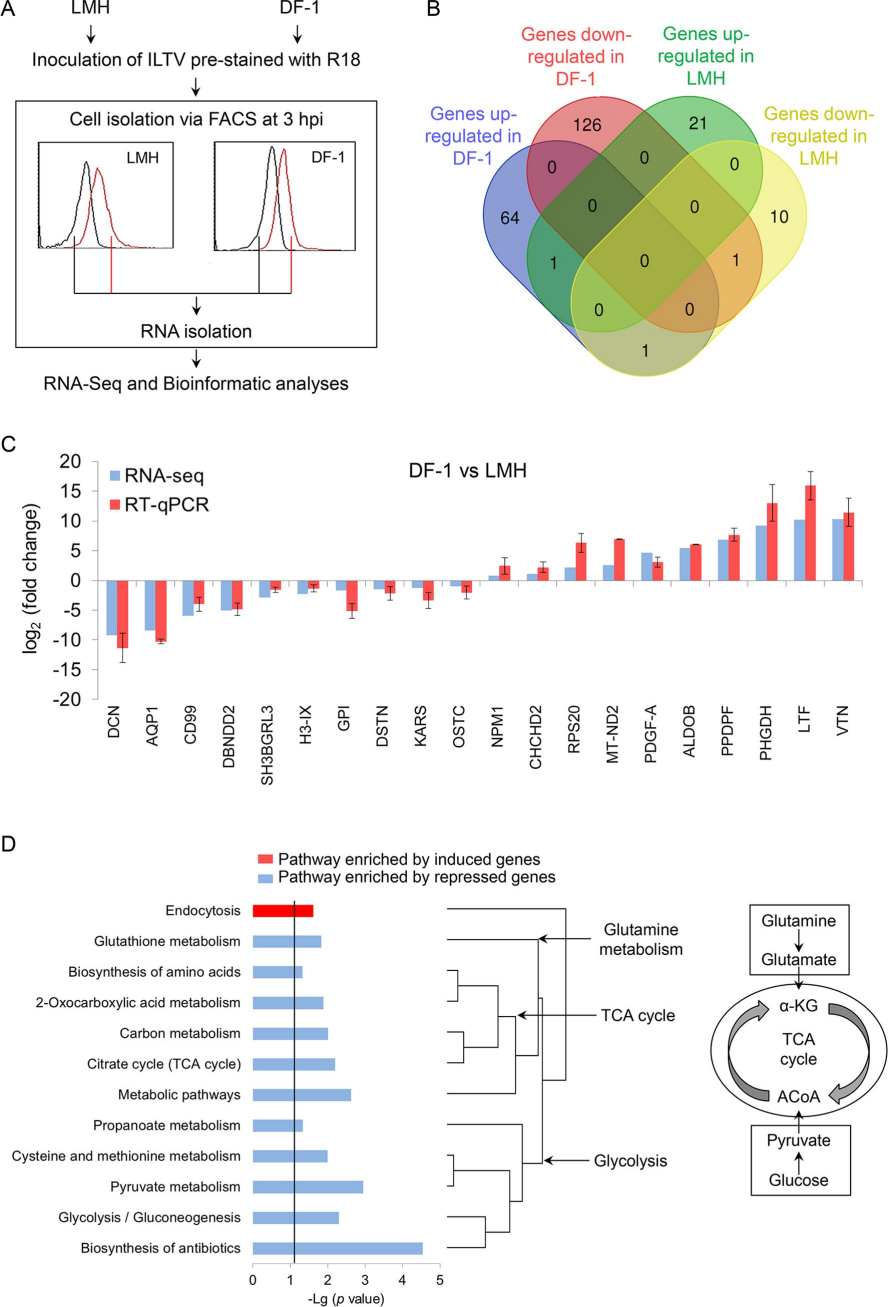
Genome-wide transcriptome analysis. (A) Workflow of genome-wide transcriptome analysis (MOI 1). (B) Venn diagram showing the intersections of genes significantly regulated by ILTV infection in LMH cells and DF-1 cells at *p*-value < 0.01, *q*-value < 0.001, and fold-change > 1.5 or < 0.667. (C) The transcriptional levels of 20 genes randomly selected from each gene set that were regulated at different levels were examined by RT-qPCR and compared with RNA-seq data for validation. The results are presented as the mean ± SD, n = 3. (D) Pathway analysis with genes significantly altered by ILTV infection in DF-1 cells was performed with a *p*-value < 0.05.

To explore the molecular mechanism underlying the distinct regulation of viral transcription in LMH cells and DF-1 cells, global transcriptome analysis of ILTV infected cell lines was conducted, as shown in Fig 3A. RNA isolated from mock-infected cells or ILTV infected cells at 3 hpi were submitted for RNA-sequencing. Bioinformatics analyses identified 66 upregulated genes and 127 downregulated genes in DF-1 cells upon ILTV infection based on the following criteria: (i) *p*-value < 0.01, (ii) *q*-value < 0.001, and (iii) fold-change > 1.5 or < 0.667 (S1 Table). Only 22 genes were upregulated, and 12 genes were downregulated in LMH cells upon ILTV infection (S1 Table). There was only 1 gene commonly regulated and 1 gene oppositely regulated between LMH cells and DF-1 cells, which demonstrated the distinct transcriptional profiles induced by ILTV infection in these two cell lines (Fig 3B). For validation purposes, the transcription levels of 20 genes randomly selected from each gene set that were regulated at different levels were examined using RT-qPCR. The results corresponded with results from the RNA-seq analysis (Fig 3C). Further pathway analysis using DAVID functional annotation with a *p*-value < 0.05 revealed that 11 pathways were significantly enriched for genes downregulated by ILTV infection and 1 pathway was significantly enriched for genes upregulated by ILTV infection in DF-1 cells (Fig 3D). We did not observe any pathway enrichment for genes altered by ILTV infection in LMH cells. It is worth noting that all pathways enriched for downregulated genes in DF-1 cells were cellular metabolism-related pathways. Most of them are involved in TCA cycle-related metabolic processes, indicating that these metabolic processes might be essential for ILTV transcription.

### Dynamics of the metabolome of DF-1 cells and LMH cells during ILTV infection

According to the transcriptome analysis, the dynamics of the metabolome of DF-1 cells and LMH cells before and after ILTV infection were investigated by untargeted metabolomics analysis using liquid chromatography mass spectrometry (LC-MS). Cell samples were harvested at 3, 9, and 24 hpi. A total of 24,106 precursor molecules, including amino acids, carbohydrates, cofactors, lipids, nucleotides, peptides, secondary metabolites, hormones, etc., in positive and negative ion mode were assayed. After ILTV infection, the levels of many metabolites were increased in DF-1 cells at 3 hpi but reduced back to normal levels at 9 hpi (S1 Fig, S2 Table). This transient increase in cell metabolism was consistent with the repression of cell metabolic pathways at 3 hpi (Fig 3D) and reflects the metabolic requirements for viral transcription in DF-1 cells, mainly including metabolites involved in glucose metabolism, glutamine metabolism, the TCA cycle, and nucleotide metabolism.

**Fig 4:**
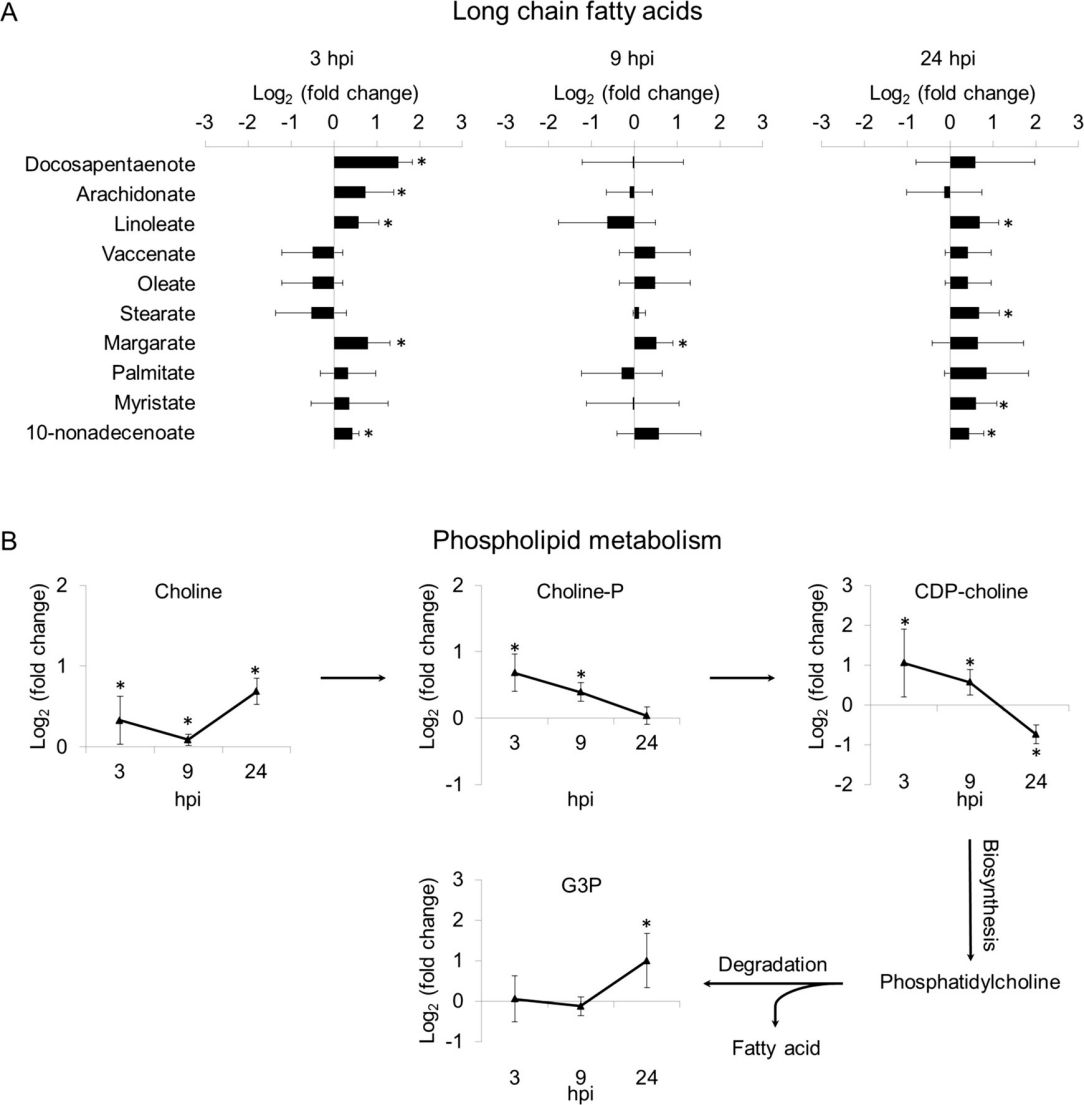
ILTV infection induces *de novo* fatty acid synthesis in DF-1 cells. DF-1 cells mock-infected or virally infected at a MOI of 1 with ILTV were harvested at 3, 9, and 24 hpi and processed for LC-MS. (A) The effects of ILTV infection on the relative levels of long chain fatty acids. (B) The effects of ILTV infection on the relative levels of metabolites involved in phospholipids metabolism. Average fold changes in the metabolite levels (relative to the mock-infected samples) of four independent biological experiments are plotted on a log_2_ axis. The results are presented as the mean ± SD (n = 4). Asterisks indicate statistical difference (*p* < 0.05). (Choline-P: choline phosphate; CDP-choline: cytidine diphosphate choline; G3P: glyceol-3-phosphate).

Glycolysis and the TCA cycle form the backbone of central carbon metabolism in cells. These two pathways either oxidize glucose to form ATP and NADH to provide energy for the cell or convert glucose to precursors of amino acids, lipids and nucleotides. The levels of glycolytic intermediates were altered in a strikingly different manner between DF-1 cells and LMH cells during ILTV infection. After ILTV infection, many metabolites of glycolysis upstream of pyruvate, namely, hexose-P, 3PG, PEP, and acetyl-CoA, were induced, while the levels of metabolites from citrate to succinate in the TCA cycle were basically not affected by infection, indicating that the promoted glucose metabolism by ILTV infection did not contribute to an increased activity of the TCA cycle (S1 Fig). ILTV might drive a shift from glycolysis to FA synthesis through a pyruvate/citrate shuttle, similar to the metabolic patterns of cells infected by HCMV [21], since both the levels of acetyl-CoA and OAA were significantly increased. This was further evidenced by the significant increases in half of the long chain fatty acids (LCFA) at 3 hpi as shown in Fig 4A. Further, the observed elevation in LCFA most likely due to the increased lipid synthesis rather than the degradation of phospholipids, since the increase was only observed in fatty acid precursor metabolites but not in glycerol-3-phosphate (G3P), metabolite associated with fatty acid production due to degradation of phospholipids (Fig 4B). The stable total level of citrate may be due to a dynamic balance between citrate generation from acetyl-CoA in mitochondria and citrate consumption for fatty acid synthesis in the cytoplasm (S1 Fig). The increased level of 6-phosphogluconate indicated an enhancement of the pentose phosphate pathway. Similar to what has been observed for HCMV infection [4], purine metabolism, which has multiple biological functions, remained stable in DF-1 cells upon ILTV infection (S1 Fig).

Glutamine is a nonessential but important amino acid for many cellular metabolic pathways, such as glutathione production, ammonia production, and nucleotide synthesis. In addition, glutamine is an alternative carbon source for the TCA cycle through a critical metabolic process called glutaminolysis by which glutamine is converted to glutamate by glutaminase and then to α-ketoglutarate (α-KG) and is frequently used by tumor cells and virally infected cells to replenish the TCA cycle when glucose carbon is shunted away from the TCA cycle. As shown in S1 Fig, both the levels of glutamine and glutamate were increased at 3 hpi by ILTV infection in DF-1 cells. However, no enhancement of α-KG and its downstream intermediates succinyl-CoA and succinate was observed. Together with the extensive induction of pyrimidine metabolism, ILTV infection most likely uses glutamine for the synthesis of nucleotides rather than replenishing the TCA cycle. Considering that most of the intermediates of the TCA cycle were not affected by ILTV infection, the enhanced level of aspartate might be supplied by the increased OAA directly synthesized from pyruvate. The stable level of pyruvate might be due to its substantial consumption to form alanine and acetyl-CoA. The recovery of normal cell metabolism after 3 hpi in DF-1 might be the consequence of either the activation of the innate immune defense system upon infection or host autofeedback mechanisms, which maintain cellular metabolic homeostasis in response to dramatic shifts in cellular metabolism.

Overall, the above analysis of the cellular metabolome of DF-1 cells during ILTV infection indicates that ILTV is most likely to direct cell glycolysis for both FA and nucleotide synthesis and utilizes cell glutamine for nucleotide synthesis but has little effect on the cell TCA cycle in general. However, this prediction is difficult to validate by monitoring the dynamics of the metabolome of LMH cells directly. As shown in S2 Fig and S2 Table, little effect of ILTV infection on metabolism could be observed in LMH cells at 3 hpi and 9 hpi, which is consistent with the finding that ILTV infection had no significant effect on the transcriptional profile of LMH cells at 3 hpi (Fig 3). This can be explained by the higher basal metabolic level of LMH cells compared with that of DF-1 cells (S3 Fig, S2 Table), which makes LMH cells insensitive to the metabolism and energy consumption at the early stage of infection. For validation purposes, detailed investigations of the effects of glycolysis, glutamine metabolism, and fatty acid synthesis on ILTV infection were then conducted in the ILTV permissive cell line LMH.

### Effects of glycolysis on ILTV infection in LMH cells

**Fig 5:**
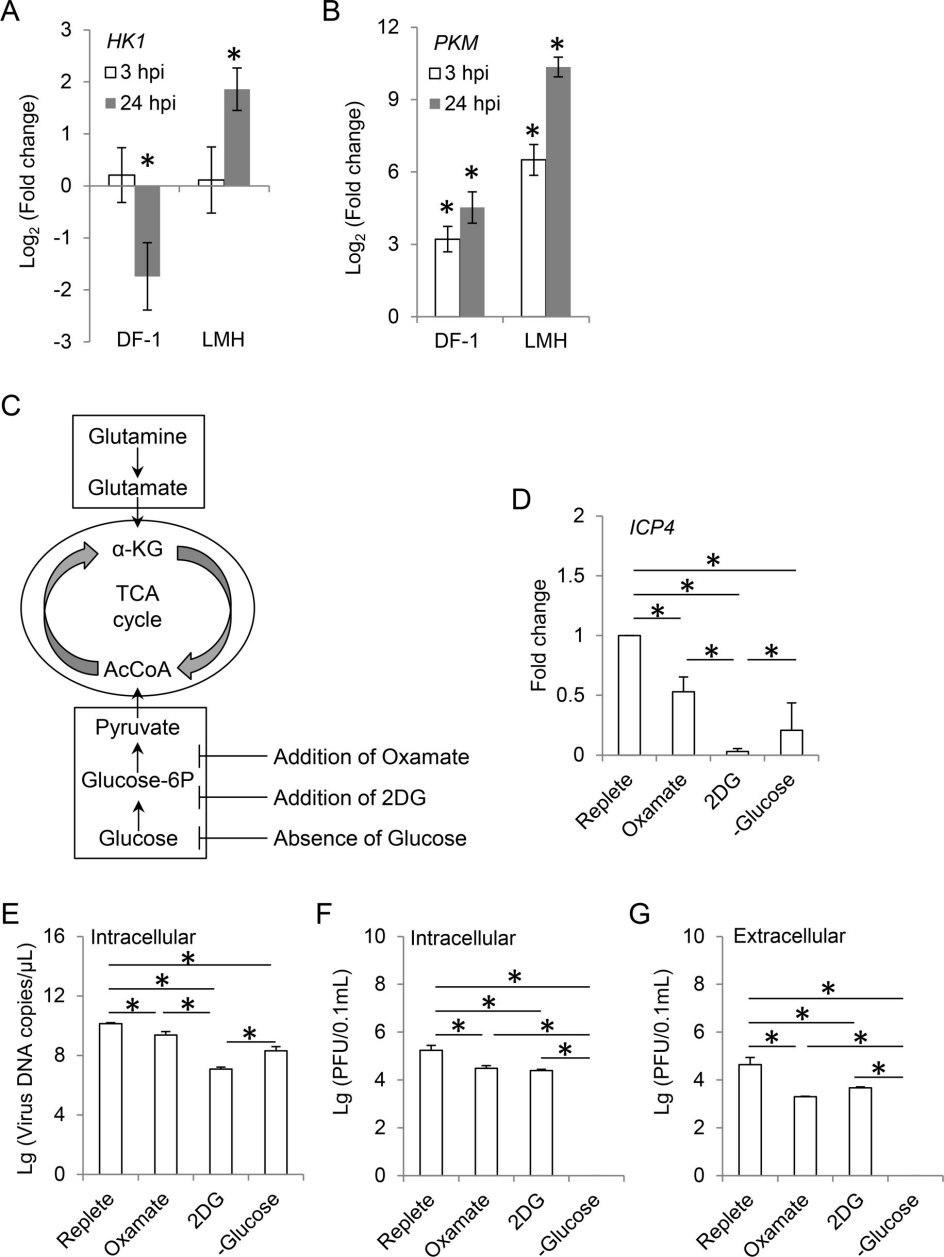
Effect of glycolysis on ILTV infection. (A and B) The effect of ILTV infection on the transcription of two key enzymes of glycolysis, hexokinase-1 (HK1) and pyruvate kinase PKM were detected by RT-qPCR in DF-1 and LMH cells at indicated time points (MOI 1). (C) Schematic presentation showing the experimental design. LMH cells were cultured in the absence of glucose or overlaid with replete medium in the presence of different pharmacological inhibitors of glycolysis, namely the inhibitor of enzyme lactate dehydrogenase Oxamate (100 mM) and the synthetic glucose analog 2-DG (50 mM). (D) The effect of glycolysis on the transcription of *ICP4* was detected by absolute RT-qPCR at 12 hpi (MOI 1). (E-G) The effects of glycolysis on viral genome replication and virion production were determined by ILTV-specific qPCR (E) and plaque assays (F and G), respectively, at 48 hpi (MOI 1). The results of statistical analyses are presented as the mean ± SD, n = 3. Asterisks indicate statistical difference (*p* < 0.05).

It is worth noting that the intracellular level of glucose was constantly enhanced by ILTV infection in DF-1 cells (S1 Fig). However, the level of its downstream intermediate hexose-P was only enhanced at 3 hpi and dropped back to normal level at 9 hpi, indicating the presence of a bottleneck at the first step of glycolysis. Therefore, the level of hexokinase-1 (HK1) was detected by RT-qPCR, which showed that the transcriptional level of *HK1* was significantly repressed in DF-1 cells but induced in LMH cells at 24 hpi (Fig 5A). In addition, the levels of both the upstream and downstream intermediates of pyruvate (PEP, alanine, acetyl-CoA, and OAA) were enhanced by ILTV infection at 3 hpi in DF-1 cells, while the level of pyruvate remained unchanged (S1 Fig). This was more likely due to the large consumption of pyruvate for multiple pathways rather than the blocking of pyruvate generation since the transcription level of pyruvate kinase PKM, which converts PEP to pyruvate, was significantly induced in both DF-1 cells and LMH cells upon ILTV infection (Fig 5B).

We next examined whether glycolysis is a required metabolic pathway for efficient viral infection of LMH cells by removing glucose from the culture medium (Fig 5C). Compared with cells cultured in replete medium, glucose deprivation significantly reduced the transcription level of *ICP4* as assayed by RT-qPCR at 12 hpi (Fig 5D) and viral genome replication as assayed by ILTV-specific qPCR at 48 hpi (Fig 5E). Strikingly, virion production was completely blocked by the absence of glucose since neither the formation of virions in cells nor the release of virions into the medium was observed (Fig 5F and 5G). Next, we addressed if the entry of glycolysis intermediates into the TCA cycle is required for ILTV replication. A synthetic glucose analog 2-deoxy-D-glucose (2DG) and oxamate, an inhibitor of the lactate dehydrogenase (LDH) enzyme, were employed in the current study to block glycolysis (Fig 5C). The administration of oxamate reduced the transcription of *ICP4* and viral genome replication to levels similar to those in cells cultured in glucose-free medium (Fig 5D and 5E). The administration of 2DG induced the greatest levels of repression of both *ICP4* transcription and viral genome replication compared with those induced by oxamate treatment and glucose deprivation (Fig 5D and 5E). However, despite being significantly reduced, virion production was not completely inhibited by blocking glycolysis with either inhibitor (Fig 5F and 5G).

Taken together, the results demonstrate that glycolysis is required for efficient ILTV infection in LMH cells.

### Effects of glutamine metabolism on ILTV infection in LMH cells

**Fig 6:**
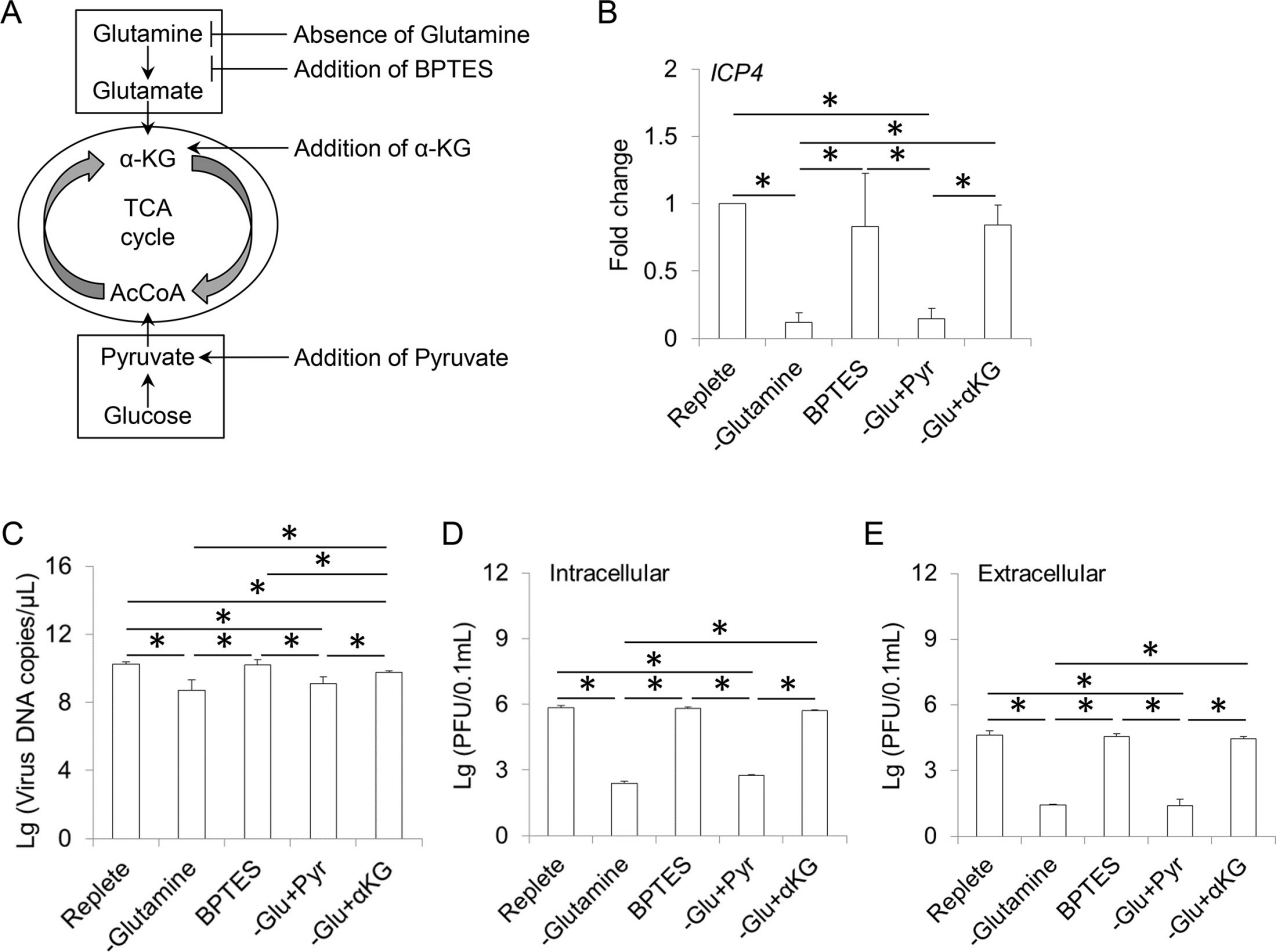
Effect of glutamine metabolism on ILTV infection. (A) Schematic presentation showing the experimental design. LMH cells were cultured in glutamine-free medium in the presence or absence of two TCA cycle intermediates, namely α-KG (5 mM) and pyruvate (Pyr, 8 mM), or overlaid with replete medium in the presence of glutaminolysis inhibitor BPTES (3 μM). (B) The effect of glutamine metabolism on the transcription of *ICP4* was detected by absolute RT-qPCR at 12 hpi (MOI 1). (C-E) The effects of glutamine metabolism on viral genome replication and virion production were determined by ILTV-specific qPCR (C) and plaque assays (D and E), respectively, at 48 hpi (MOI 1). The results of statistical analyses are presented as the mean ± SD, n = 3. Asterisks indicate statistical difference (*p* < 0.05).

To verify whether glutamine metabolism is necessary for ILTV replication in host cells, viral transcription, viral genome replication and virion production were measured in LMH cells in the presence or absence of glutamine (Fig 6A). RNA from each sample was isolated at 12 hpi, and viral transcription was detected via RT-qPCR. Without the addition of glutamine to cell cultures, the transcription level of *ICP4* decreased significantly (Fig 6B), followed by the subsequent reduction in viral genome replication (Fig 6C) and virion production (Fig 6D and 6E). However, the effects of glutamine on ILTV replication were not altered by the addition of the glutaminolysis inhibitor Bis-2-(5-phenylacetamido-1,2,4-thiadiazol-2-yl) ethyl sulfide (BPTES), which was consistent with the unchanged level of α-KG during ILTV infection (S1 Fig), suggesting that glutamine contributes to ILTV replication through nucleotide biosynthesis directly rather than through anaplerosis, which fuels the TCA cycle via α-KG. This conclusion was further supported by the finding that the addition of pyruvate failed to restore viral replication in the absence of glutamine. However, supplementation with α-KG partially rescued viral replication in the absence of glutamine (Fig 6B–6E), which might be due to the biosynthesis of glutamine from glutamate mediated by α-KG. Taken together, the above findings demonstrated that glutamine metabolism is required for the maximal replication of ILTV in LMH cells in ways independent of glutaminolysis.

### Effects of fatty acid synthesis on ILTV infection in LMH cells

**Fig 7:**
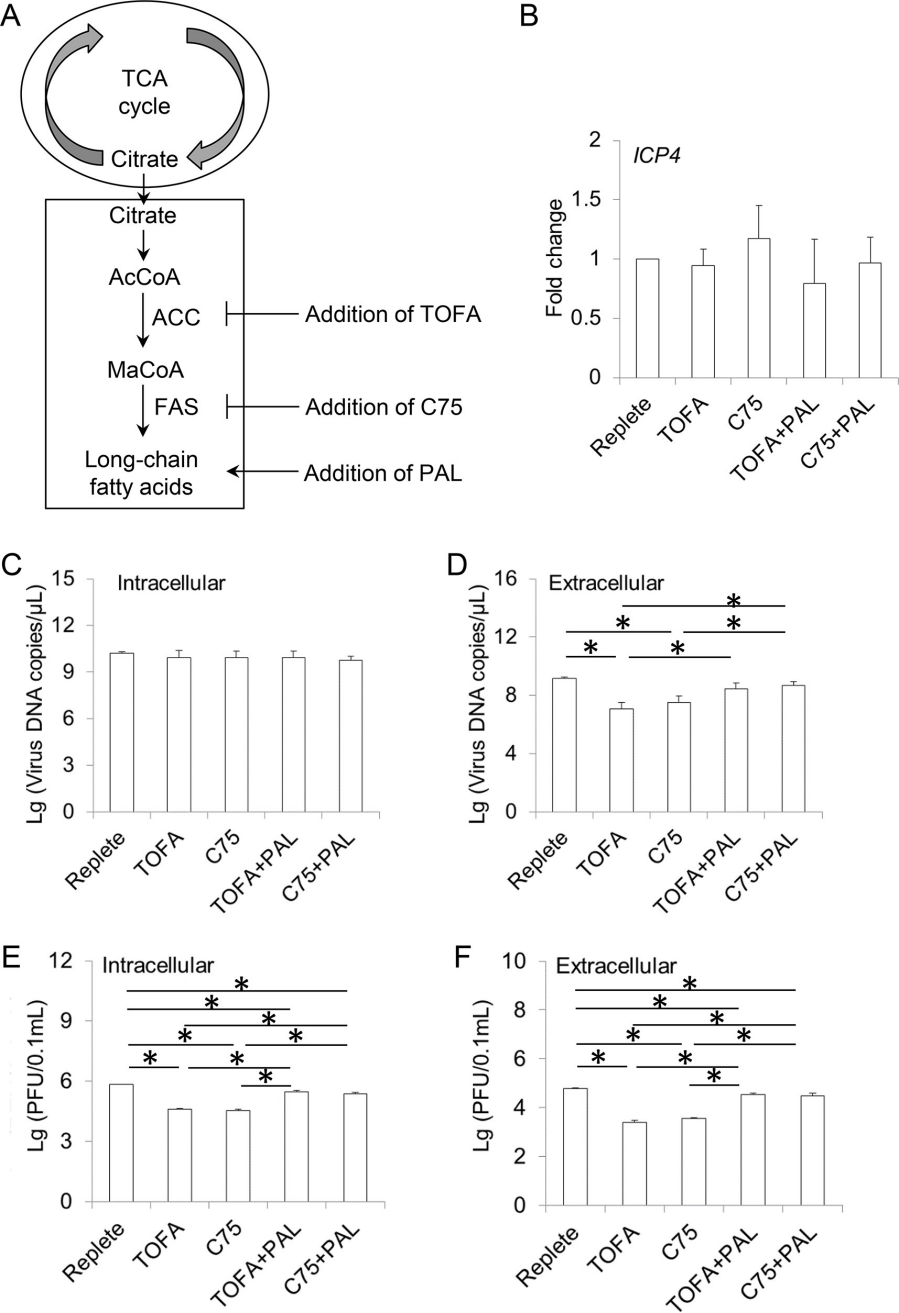
Effect of fat acid synthesis on ILTV infection. (A) Schematic presentation showing the experimental design. LMH cells were cultured in replete medium in the presence or absence of two inhibitors of fat acid synthesis, namely acetyl-CoA carboxylase inhibitor TOFA (20 μg/mL) and fatty acid synthase inhibitor C75 (10 μM) with or without the supplementation of palmitic acid (PAL, 32 mM). (B) The effect of fat acid synthesis on the transcription of *ICP4* was detected by absolute RT-qPCR at 12 hpi (MOI 1). (C-F) The effects of glutamine metabolism on viral genome replication and virion production were determined by ILTV-specific qPCR (C and D) and plaque assays (E and F), respectively, at 48 hpi (MOI 1). The results of statistical analyses are presented as the mean ± SD, n = 3. Asterisks indicate statistical difference (*p* < 0.05).

Our metabolome analysis indicated that fatty acids might act as a sink for carbon flowing from glycolysis through the citrate transport system (S1 Fig). To address the role of FA synthesis from the citrate transport system in ILTV infection, an allosteric inhibitor of acetyl-CoA carboxylase-α (ACC) called 5-tetradecyloxy-2-furoic acid (TOFA), which blocks malonyl-CoA synthesis, and the fatty acid synthase (FAS) inhibitor C75 were employed to block this process (Fig 7A). Neither TOFA nor C75 had any effect on viral transcription (Fig 7B) or subsequent viral genome replication (Fig 7C), as evidenced by the assays measuring *ICP4* transcription and intracellular viral genome DNA copy numbers by RT-qPCR and ILTV specific RT-qPCR, respectively. However, the number of extracellular viral genomic DNA was reduced by both inhibitors and could be rescued via the addition of palmitate (PAL) (Fig 7D). This finding was consistent with the repressed levels of both intracellular and extracellular infectious viral particles (Fig 7E and 7F). Thus, viral genome replication was not affected by cell FA biosynthesis, and the decrease in the extracellular level of viral genomic DNA was likely due to the reduced amount of extracellular infectious viral particles. Although both intracellular virion production and the release of virions from the host cell could affect extracellular virion levels, the reduced level of extracellular virions was most likely due to the former since FA biosynthesis affected intracellular virion production in the same way as the extracellular levels of viral DNA and virions (Fig 7E and 7F). Thus, the above data suggest that FA biosynthesis is required for intracellular virion production but has no effect on viral transcription and viral genome replication.

### Validation of the metabolic requirements of ILTV infection in DF-1 cells and primary cells

**Fig 8:**
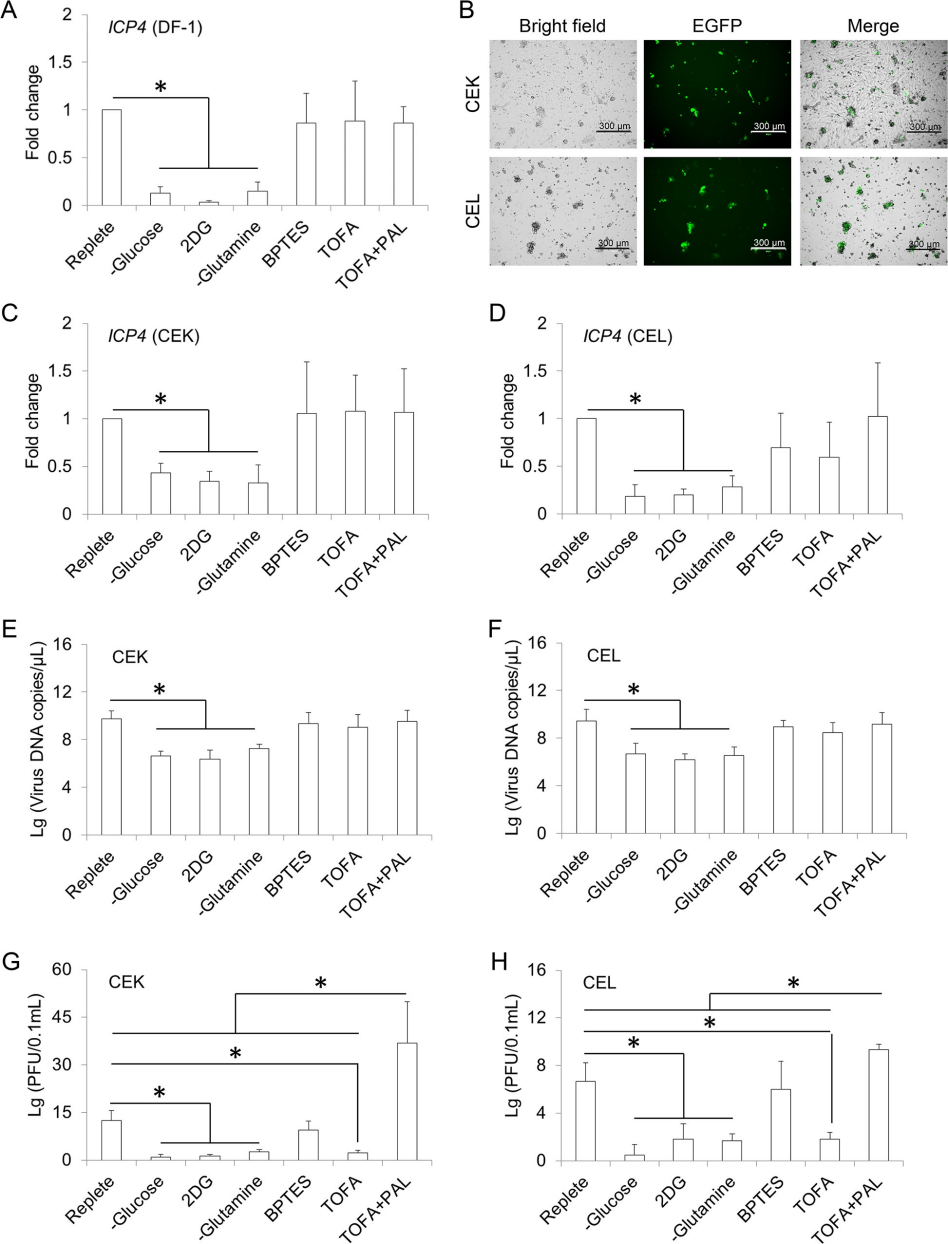
Validation of the metabolic requirements of ILTV infection in DF-1 cells and primary cells. (A) DF-1 cells were cultured in the absence of glucose or glutamine or overlaid with replete medium in the presence or absence of glycolysis inhibitor 2-DG (50 mM), glutaminolysis inhibitor BPTES (3 μM), or fat acid synthesis inhibitor TOFA (20 μg/mL) with or without the supplementation of palmitic acid (PAL, 32 mM). The transcription of *ICP4* was detected by absolute RT-qPCR at 3 hpi (ILTV, MOI 1). The results of statistical analyses are presented as the mean ± SD, n = 4. Asterisks indicate statistical difference (*p* < 0.05). (B-H) Primary chicken embryo kidney cells (CEK) and chicken embryo livers cells (CEL) were isolated from 19-day-old specific pathogen-free (SPF) chicken embryos. (B) CEK and CEL were infected with ILTV-EGFP strain at a MOI 5. The infection of ILTV-EGFP was detected by fluorescence microscopy via tracing the EGFP signal at 24 hpi. Representative figures of ILTV-infected cells are shown. The scale bar indicates 300 μm. (C-H) CEK (C, E, and G) and CEL (D, F, and H) were cultured in the absence of glucose or glutamine or overlaid with replete medium in the presence or absence of glycolysis inhibitor 2-DG (50 mM), glutaminolysis inhibitor BPTES (3 μM), or fat acid synthesis inhibitor TOFA (20 μg/mL) with or without the supplementation of palmitic acid (PAL, 32 mM). (C and D) The transcription of *ICP4* was detected by absolute RT-qPCR at 6 hpi (ILTV, MOI 1). (E-H) Viral genome replication and virion production were determined by ILTV-specific qPCR (E and F) and plaque assays (G and H), respectively, at 48 hpi (ILTV, MOI 1). The results of statistical analyses are presented as the mean ± SD, n = 4. Asterisks indicate statistical difference (*p* < 0.05).

The importance of cell glycolysis and glutamine metabolism to viral gene transcription was further evidenced in DF-1 cells, since the transcription level of *ICP4* was significantly decreased by glucose deprivation, glutamine deprivation, and the administration of 2DG in DF-1 cells upon ILTV infection (Fig 8A). While, similar to what we revealed in LMH cells, neither the administration of BPTES nor the alteration of fatty acid metabolism using TOFA and PAL had any effect on *ICP4* transcription (Fig 8A). To determine whether the metabolic requirements of ILTV infection revealed in chicken cell lines also exist in primary cells, further validations were performed in primary cells isolated from specific pathogen free (SPF) chicken embryos, including chicken embryo kidney cells (CEK) and chicken embryo liver cells (CEL). An ILTV-LSJ09 strain expressing enhanced green fluorescent protein (EGFP), which exhibits similar viral replication kinetics and CPEs of infection as the wild type stain [13, 15], was employed to monitor viral infection in CEK and CEL. Both CEK and CEL comprised a heterogeneous population of cells, which could be divided morphologically into fibroblast-like cells and non-fibroblast like cells (S4 Fig and Fig 8B). To ensure that all cells in the heterogeneous population were efficiently infected, primary cells were infected with the ILTV-EGFP strain at a MOI of 5. Upon infection with this EGFP strain, EGFP signal was observed in both primary cultures but only presented in the non-fibroblast like cells (Fig 8B). Compared with cells cultured in replete medium, either glucose deprivation, glutamine deprivation, or the administration of 2DG significantly reduced the transcription level of *ICP4* at 6 hpi (Fig 8C and 8D), followed by the subsequent reduction in viral genome replication (Fig 8E and 8F) and virion production at 48 hpi (Fig 8G and 8H). Different from what we observed in LMH cells (Fig 5F and 5G), the administration of 2DG repressed virion production as efficiently as glucose deprivation in primary cells, indicating that primary cells might be more sensitive to 2DG than the tumor cell line LMH. No effect of the administration of BPTES on any step of viral replication was observed in these primary cells (Fig 8C–8H). Similar to the results of studies in cell lines, the administration of TOFA had no effect on either *ICP4* transcription (Fig 8C and 8D) or viral genome replication (Fig 8E and 8F) and only reduced virion production significantly in primary cultures (Fig 8G and 8H). Notably, the primary cells seemed more sensitive to long-chain saturated fatty acid than LMH cells, since the addition of PAL not only rescued the virion production reduced by TOFA but also increased the virion production to the levels significantly higher than those of cells cultured in replete medium (Fig 8G and 8H).

Overall, our metabolome analysis in combination with functional validations demonstrates that ILTV infection tends to promote a metabolic shift from glycolysis to FA and nucleotide synthesis and utilizes glutamine in ways independent of glutaminolysis, without an obvious general effect on the TCA cycle in host cells. Both glucose and glutamine are required for the transcription of viral genes and subsequent steps of viral replication. However, FA synthesis is essential for virion formation but not required for other upstream steps of ILTV replication.

## Discussion

**Fig 9:**
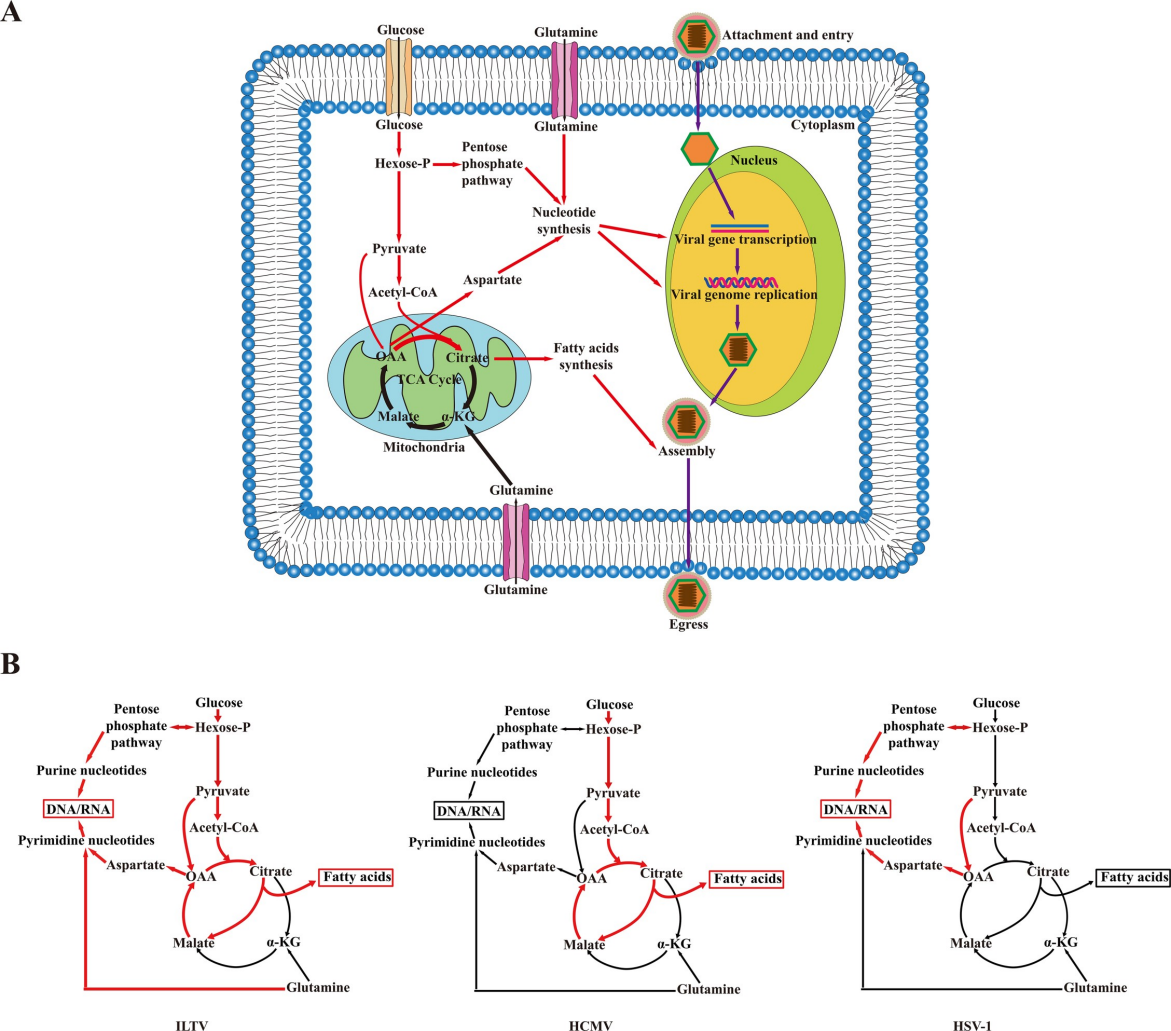
A speculative model summarizing the metabolic requirements of ILTV replication. (A) Glycolysis, glutamine metabolism, and fatty acid synthesis are required for different steps of ILTV replication. Both glycolysis and glutamine metabolism are required for early viral gene expression, while fatty acid synthesis contributes to viral assembly and viral egress but not viral gene transcription. (B) Divergent effects of ILTV, HCMV and HSV-1 on central carbon metabolism. Schematic summary of the major host metabolite concentration changes and speculative flux changes in response to ILTV (left panel) according to the findings of present study and the metabolite concentration and flux changes in response to HCMV (middle panel) and HSV-1 (right panel) infection proposed by previous study [2]. Arrow colors denote flux changes (red, metabolic flux promoted by ILTV infection; black, no effect). (Hexose-P: glucose-6-phosphate and its isomers; α-KG: α-ketoglutarate; OAA: oxaloacetate).

Recently, host cell metabolism has been considered as a promising target for therapeutics against herpesviruses. However, the comprehensive exploration of the metabolic requirements of herpesvirus replication has only been applied for a few human herpesviruses, namely, HCMV, VZV, KSHV, HSV, and EBV. The metabolic requirements of other herpesviruses, especially animal herpesviruses, remain largely unclear. Gallid alphaherpesvirus 1 (ILTV) is an important animal herpesvirus that continues to cause substantial economic losses to the poultry industry worldwide and thought to be an early type of alphaherpesviruses [9]. Here, using the ILTV-susceptible but nonpermissive chicken cell line DF-1 and the permissive chicken cell line LMH as models, we performed the first metabolome analysis of an animal herpesvirus and revealed several characteristics of the metabolic requirements of ILTV. As shown in Fig 9, different cell metabolic pathways are required at distinct stages of ILTV replication. Both glucose and glutamine are required for viral transcription, which influences subsequent viral genome replication and assembly steps. Although unnecessary for viral transcription and viral genome replication, FA synthesis is essential for the assembly and egress of ILTV. Interestingly, ILTV infection promotes cell glucose and glutamine metabolism but maintains the cell TCA cycle at a stable state. It is likely that ILTV infection promotes the metabolic flux from glucose towards three directions that include *de novo* nucleotide synthesis via the pentose phosphate pathway, generation of aspartate by replenishing OAA directly from pyruvate, and FA biosynthesis through a pyruvate/citrate shuttle. Moreover, ILTV infection utilizes glutamine independently of glutaminolysis, which may be brought about through the promotion of metabolic flux from glutamine to nucleotide synthesis directly without obvious effect on the anaplerotic entry of glutamine into the TCA cycle. Previous studies, including ours, have revealed that ILTV infection raises the threshold of infected host cells for infection-induced host cell death, which prolongs ILTV replication in infected cells and allows the infiltrated virus to achieve optimal replication [10–12]. Therefore, the consumption of glucose and glutamine in ways with little interference with the cell TCA cycle might be an evolved strategy by which ILTV maintains a balance between infection and host cell survival.

Cellular metabolic requirements for maximal viral production of human herpesviruses have been examined for HCMV, HSV-1, and KSHV [2, 4, 5, 21, 22], which suggests that, although similarities exist, these human herpesviruses have divergent effects on cellular metabolism. For example, HCMV tends to enhance the glycolytic flux to fuel fatty acid biosynthesis by generating related TCA metabolites, while HSV-1 tends to deliver the carbon resources from glucose to pyrimidine synthesis via pyruvate carboxylase. Neither requires glutamine for efficient replication. ILTV combines the metabolic characteristics of HCMV and HSV-1 and also requires glutamine for efficient viral replication (Fig 9), indicating that multiple metabolic pathways could be targeted simultaneously to achieve more effective therapy for ILTV infection. KSHV requires cellular glucose, glutamine, and FA for its replication at distinct stages [22]. Glycolysis is required for the transcription of immediate-early genes, glutaminolysis is required for the transcription of early genes, and FA synthesis is required for virion assembly and egress. Similar to KSHV, ILTV requires the same resources for viral replication but some differences were revealed. In addition to glucose, glutamine is also required for the transcription of the *ICP4* immediate-early gene of ILTV. This difference might be due to the distinct shifts in the usage of glutamine whereby KSHV consumes glutamine through glutaminolysis, while ILTV may direct glutamine to nucleotide synthesis directly without entering the TCA cycle (Fig 9). These findings confirm the current view that the metabolic requirements of different herpesviruses are very virus specific. However, the investigation of metabolic interactions between herpesviruses and their hosts is still in its infancy, and the metabolic requirements of most herpesviruses remain unclear. Therefore, extensive investigations of the metabolic requirements of more herpesviruses are urgently needed to see whether any high conservation of metabolic program within the same subfamily exists and to refine the rational design of virus-specific therapeutics for the treatment of herpesviruses.

It is worth noting that, despite the transient promotion of metabolic processes (S1 Fig), the transcription of metabolic genes were significantly repressed in DF-1 cells at 3 hpi (Fig 3D). It might be the consequence of either the activation of the innate immune defense system upon infection or host auto feedback mechanism that maintains cellular metabolic homeostasis in response to dramatic shifts in cellular metabolism. Given the importance of these metabolic processes in ILTV infection (Figs 5–8), the repression of these metabolic pathways might be part of the reasons why DF-1 cells only support transient transcription of viral genes but not further viral replication. Further investigation was needed to elucidate the molecular mechanism determining the nonpermissivity of DF-1 cells to ILTV infection.

All herpesviruses, including ILTV, establish latency after acute infection and cannot be cleared from the host currently; therefore, reactivation from latent infection frequently occurs when the host is under stress or host immunity is compromised [9, 23–26]. Previous studies of KSHV have demonstrated that the three metabolic pathways required for viral replication are also essential for the survival of endothelial cells latently infected by KSHV, indicating the possibility of developing drugs targeting both latent and lytic infection to cure KSHV infection [5, 22]. Although these metabolic pathways are also required for ILTV replication (Fig 9), the effects of blocking these pathways on ILTV latency and reactivation remain unclear. Due to the lack of a reliable *in vitro* model for latent infection of ILTV, *in vivo* studies in chickens infected with ILTV upon the administration of different metabolic inhibitors may help to answer this question.

Based on the results obtained using different metabolic regulators, we demonstrate that ILTV infection promotes cell glucose and glutamine metabolism but maintains the cell TCA cycle at a stable state. We further proposed a model of the manipulation of cell metabolism by ILTV infection according to later metabolome analysis, which suggests that ILTV infection most likely promotes the metabolic flux from glucose to *de novo* nucleotide biosynthesis via the pentose phosphate pathway, the generation of aspartate by replenishing OAA directly from pyruvate, and FA biosynthesis through a pyruvate/citrate shuttle (Fig 9). Additional metabolic flux tracing methods are needed to investigate the exact metabolic flux and strength of the model we proposed here.

## Materials and methods

### Ethics statement

The isolation of primary cells from chicken embryos was approved and performed in accordance with the ethical guidelines of the Animal Ethics Committee of Harbin Veterinary Research Institute of the Chinese Academy of Agricultural Sciences (approval no. SYXK (Hei) 2011022).

### Viral strain and cells

The virulent ILTV-LJS09 strain (GenBank Accession No. JX458822) is stored at the Harbin Veterinary Research Institute of CAAS. This strain was isolated in 2009 from 6-week-old Yellow-Foot layers housed on a farm in Jiangsu Province, China, which had not been vaccinated against ILTV, and its genome has been sequenced. This strain can be propagated in a chemically immortalized leghorn male hepatoma (LMH) cell line with clear CPEs observed [27, 28]. An ILTV-LSJ09 strain expressing enhanced green fluorescent protein (EGFP), which exhibits no significant difference from the wild type in either viral replication or the CPEs of infection as described previously [13, 15], is stored at the Harbin Veterinary Research Institute of CAAS. The chicken fibroblast cell line DF-1 (ATCC CRL-12203) and the chicken hepatoma cell line LMH (ATCC CRL-2117) were maintained in Dulbecco’s modified Eagle’s medium (DMEM) supplemented with 10% fetal bovine serum (FBS), 100 units/ml penicillin, 100 μg/ml streptomycin and 2 mM L-glutamine. Cell cultures were incubated at 37°C in 5% CO_2_. Primary chicken embryo kidney cells (CEK) and chicken embryo livers cells (CEL) were isolated from 19-day-old specific pathogen-free (SPF) chicken embryos (Harbin Weike Biotechnology Development Co., Ltd., Harbin, China). The kidneys and livers were harvested, homogenized, and then incubated in a 0.25% trypsin solution for 15 min at 37°C. Cells dissociated from kidneys and livers were suspended in a 1:1 ratio of DMEM supplemented with 0.45% glucose plus 10% FBS, 100 units/ml penicillin, 100 μg/ml streptomycin, and 2 mM L-glutamine. Cell suspensions were filtered through 70 μm nyclon cell strainer and 40 μm nyclon cell strainer (Biosharp, Hefei, China), respectively, and then centrifuged at 250 × g for 10 min at room temperature. The pellets suspended in 15 ml of DMEM supplemented with 10% FBS, 2 mM L-glutamine, 50 mM sodium bicarbonate, 100 units/ml penicillin, and 100 μg/ml streptomycin. Cell cultures were incubated at 39°C in 5% CO_2_ until cells completely attached to the bottom of the plate.

### Experimental design

To determine the susceptibility and permissibility of DF-1 cells and LMH cells to ILTV, the fusion between the viral membrane and cell membrane was detected by FACS using virus preincubated with a lipophilic dye octadecyl rhodamine B chloride (R18; Invitrogen, Carlsbad, CA), the entry and cellular distribution of ILTV were observed by electron microscopy, the insertion of viral genomes into cell nuclei and the replication of viral genomes were detected by ILTV-specific absolute quantitative real-time PCR (qPCR), the transcription of six ILTV genes covering all stages of ILTV transcription were detected by RT-qPCR, virion production was assayed by plaque assay, the effect of ILTV infection on infected cells was investigated via the detection of cytopathogenic effect (CPE) of ILTV and cell growth. Next, to explore the molecular mechanism underlying the distinct regulation of viral transcription in LMH cells and DF-1 cells, genome-wide transcriptional profiling was performed at the time point when the difference in viral gene transcription occurred between the two cell lines. According to the indication drawn by transcriptome analysis, the dynamics of the metabolome of DF-1 cells and LMH cells before and after ILTV infection were explored by untargeted metabolomics analysis using LC-MS. Finally, to verify the hypothesis of the metabolic requirements of ILTV infection that we proposed based on the metabolome analysis, functional validations in LMH cells, DF-1 cells, and primary cells were conducted via the deprivation of specific nutrient and the manipulation of specific metabolic processes in cells using chemical regulators. Detailed information was stated in the following corresponding paragraphs.

### Reagents

The synthetic glucose analog 2DG, the inhibitor of LDH Oxamate, the glutaminolysis inhibitor BPTES, pyruvate, α-KG, the allosteric inhibitor of acetyl Co-A carboxylase TOFA, the synthetic FAS inhibitor C75, and Palmitic acid (PAL) were purchased from Sigma Aldrich (Sigma Aldrich, St. Louis, MO). Given that TOFA, C75, PAL, BPTES were dissolved in dimethyl sulfoxide (DMSO, Sigma Aldrich), cells treated with DMSO at the same volumes were used as chemical control for these treatments.

### Nutrient starvation

For glucose, glutamine, and fatty acid depletion studies, DMEM lacking glucose, glutamine, pyruvate, and phenol red (Gibco, Carlsbad, CA) was used as base medium. This base medium was supplemented with 10% dialyzed FBS (Wisent, Montreal, QC, Canada), 100 units/ml penicillin, 100 μg/ml streptomycin, 1 g/liter glucose, and/or 2 mM glutamine according to the specific requirements of each experiment. The commercially available dialyzed FBS was exhaustively dialyzed against saline to ensure that glucose and glutamine had been removed. The dialyzed FBS was thoroughly tested to ensure that it maintains the ability to support cell growth under normal condition. Cells grown in replete medium (1 g/L glucose plus 2 mM glutamine) were washed three times with glutamine- or glucose-free medium and refed in the appropriate medium as needed. For functional validation experiments, 50 mM 2-DG, 100 mM sodium Oxamate, 5 mM α-KG, 3 μM BPTES, 8 mM pyruvate, 20 μg/mL TOFA, 10 μM C75, and/or 32 mM PAL were added to the replete medium according to specific requirements. No cytotoxity effect of abovementioned treatments was observed in LMH cells as assayed by trypan blue staining (S5 Fig).

### Viral quantitation and cell viability assay

LMH cells, DF-1 cells, and primary cells were infected with ILTV or ILTV-EGFP strain at multiplicity of infection (MOI) of 0.1, 1, or 5 according to the specific experimental procedures. The indicated MOI was obtained according to the number of cells to be infected and the estimated number of infectious particles, based on plaque-forming units detected in LMH cells. Levels of virus replication were determined using plaque assays and ILTV-specific qPCR assays as previously described [15]. The total level of viral replication, the cell-associated viruses, and the viruses released into supernatant were collected respectively for virus quantification. Cells were lysed via three rounds of freezing-thawing. The viability of cells was detected by trypan blue staining (Beyotime Institute of Biotechnology, Beijing, China) according to the manufacturer’s instructions.

### Fluorescence activated cell sorting (FACS)

FACS analyses were conducted using a BD FACScan cell sorter and CellQuest software version 4.0.2 (Becton Dickinson-Pharmingen, San Diego, CA). Virus and cell fusion were detected using R18 staining kit (Invitrogen) according to the manufacturer’s instructions. R18 is a lipophilic dye, which is quenched at high dye concentration but is released at dilution, and wildly used for virus penetration detection. R18 was incorporated into the lipid bilayer of ILTV at high concentration, under which condition its fluorescence is quenched. The quenched fluorescence could be recovered by the diffusion of R18 on the plasma membrane of infected cells during the membrane fusion between viruses and cells and assayed by FACA.

### Transmission electron microscopy

Samples were sent to Plague Diagnose and Technical Service Center of Harbin Veterinary Research Institute for transmission electron microscopy analysis. Briefly, cell samples were coated with gold/palladium alloy by sputter coating at 3 or 24 hpi and examined under a Hitachi H-7650 transmission electron microscope (Hitachi High Technologies, Shanghai, China), and images were taken using an AMT CCD camera (Advanced Microscopy Techniques, Woburn, MA).

### RT-qPCR

RNA was isolated from cells using EasyPure RNA Purification Kit (TransGen Biotech, Beijing, China) according to the manufacturer’s protocol. RT-qPCR and absolute RT-qPCR were performed using the SYBR PrimeScriptTM Kit (TaKaRa Bio Inc, Tokyo, Japan) as described previously [29]. The standers of the absolute RT-qPCR were prepared by cloning the PCR products of *ICP4*, *ICP27*, *UL48*, *gI*, *gG*, and *gC* genes of ILTV into the pMD18-T plasmid (TaKaRa-Bio, Shiga, Japan) according to the manufacturer’s instructions. Primer sequences are presented in S3 Table. Data was calculated with 2^-ΔΔCT^ method and results were presented as Log_2_ fold change.

### RNA sequencing

Genome-wide gene expression profiling of DF-1 and LMH cells was performed via RNA deep sequencing by Annoroad Gene Technology Co., Ltd. (Beijing, China). Four biological repeats were performed. RNA was isolated from cells using RNeasy Plus mini kit (QIAGEN, Hilden, Germany). Library construction was performed using the Illumina platform (Illumina, Inc., San Diego, CA), according to the manufacturer’s instructions. Samples were sequenced on an Illumina HiSeq 2500 instrument.

### High-throughput data analysis

RNA sequencing data was analyzed with the Galaxy web-based tool [30]. Pathway analysis was performed with DAVID (gene-enrichment analysis using EASE Score, a modified Fisher exact *p*-value, as the threshold) [31]. RNA sequencing raw data was uploaded to the National Center for Biotechnology Information database under the accession number GSE138648.

### Metabolome analysis

LMH cells and DF-1 cells with or without ILTV infection were collected at 3, 9 and 24 hpi respectively and frozen in liquid nitrogen. Four biological repeats were performed. Metabolite extraction, liquid chromatography coupled to mass spectrometry (LC-MS), and metabolome data analysis were performed by Hangzhou Lianchuan Biological Technology Co., Ltd. (LC-Bio, Hangzhou, Zhejiang province, China; www.lc-bio.com). Briefly, samples were thawed at 4°C and 100 μL of each sample was transferred into 1.5 ml centrifuge tubes. Then 200 μL of methanol (pre-cooled at -20°C) was added to each tube and vortex for 60 sec. After centrifugation for 10 min at 15,000×g at 4°C, the supernatant was transferred into another 1.5 mL centrifuge tube. Sample extracts were obtained by filtering through a 0.22 μm membrane and subjected to LC-MS system for analysis. For the preparation of quality control samples, 20 μL of extract was taken from each sample. These extracts were then mixed together and used as quality control. Chromatographic separation was accomplished in an Acquity UPLC system equipped with an ACQUITY UPLC. HSS T3 (150×2.1 mm, 1.8 μm, Waters, Milford, MA) column maintained at 40°C. The temperature of the autosampler was 4°C. Gradient elution of analytes was carried out with 0.1% formic acid in water and 0.1% formic acid in acetonitrile at a flow rate of 0.25 mL/min. The ESI-MSn experiments were executed on the Thermo LTQ-Orbitrap XL mass spectrometer with the spray voltage of 4.8 kV and 4.5 kV in positive and negative modes, respectively. Raw data was processed by the Proteowizard software (v3.0.8789) convert into mzXML format. Further analyses were performed using R (v3.3.2). Data of Figs 4, 6 and 7 are presented in S3 Table.

### Statistical analysis

The SPSS software package (SPSS for Windows version 13.0, SPSS Inc., Chicago, IL) was used for all statistical analyses. Data obtained from several experiments are reported as the mean ± standard deviation (SD). The significance of differences between two groups was determined with tow-tailed Student's t-test. One-way or two-way analysis of variances with Bonferroni correction was employed for multi-group comparison. For all analyses, a probability (*p*) value of < 0.05 was considered statistically significant.

## Supporting information

S1 Fig Schematic illustrating the metabolite changes induced by ILTV infection in DF-1 cells. DF-1 cells mock-infected or virally infected at a MOI of 1 with ILTV were harvested at 3, 9, and 24 hpi and processed for LC-MS. Average fold changes in the metabolite levels (relative to the mock-infected samples) of four independent biological experiments are plotted on a log_2_ axis (n = 4). (Hexose-P: glucose-6-phosphate and its isomers; FBP: fructose-1,6-bisphosphate; DHAP: dihydroxy acetone-phosphate; 3PG: 3-phosphoglycerate; GAP: glyceraldehyde-3-phosphate; PEP: phosphoenolpyruvate; α-KG: α-ketoglutarate; OAA: oxaloacetate; pentose-P: pentose-phosphate; PRA: 5-phosphoribosylamine; PRPP: 5-phosphoribosyl pyrophosphate.).
(TIF)

S2 Fig Schematic illustrating the metabolite changes induced by ILTV infection in LMH cells. LMH cells mock-infected or virally infected at a MOI of 1 with ILTV were harvested at 3, 9, and 24 hpi and processed for LC-MS. Plots of individual metabolite abundance during ILTV infection are the same as presented in S1 Fig.
(TIF)

S3 Fig Schematic illustrating the distinct metabolic patterns between DF-1 cells and LMH cells. DF-1 cells and LMH cells without ILTV infection were harvested and processed for LC-MS. Average fold changes in the metabolite levels (LMH relative to the DF-1) of four independent biological experiments are log_2_ transformed and presented as the font color of each metabolite according to the red-green color scale of the scheme (green, the level of indicated metabolite in LMH cells is lower than that in DF-1 cells; red, the level of indicated metabolite in LMH cells is higher than that in DF-1 cells; black, no difference between two cell lines). The abbreviations are the same as presented in S1 Fig.
(TIF)

S4 Fig Morphology of primary cells. Representative images for primary CEK and primary CEL were obtained by inverted microscopy. The scale bar indicates 300 nm.
(TIF)

S5 Fig The effects of tested reagents and treatments on cell viability in LMH cells. The viability of cells was detected by trypan blue staining according to the manufacturer’s instructions. The results are presented as the mean ± SD, n = 3. Asterisks indicate statistical difference (*p* < 0.05).
(TIF)

S1 Table List of differentially expressed genes at *p* < 0.01, *q* < 0.001, fold-change > 1.5 or < 0.667. (XLSX)

S2 Table Metabolome data presented in Fig 4 and S1–S3 Figs. (XLSX)

S3 Table List of RT-qPCR primers. (DOCX)
