# Supplementary figures and images for: Global exploration of the metabolic requirements of gallid alphaherpesvirus 1
Source: PLoS Pathog. 2020 Aug 24;16(8):e1008815. doi: 10.1371/journal.ppat.1008815 (PMC7470321; doi:10.1371/journal.ppat.1008815)

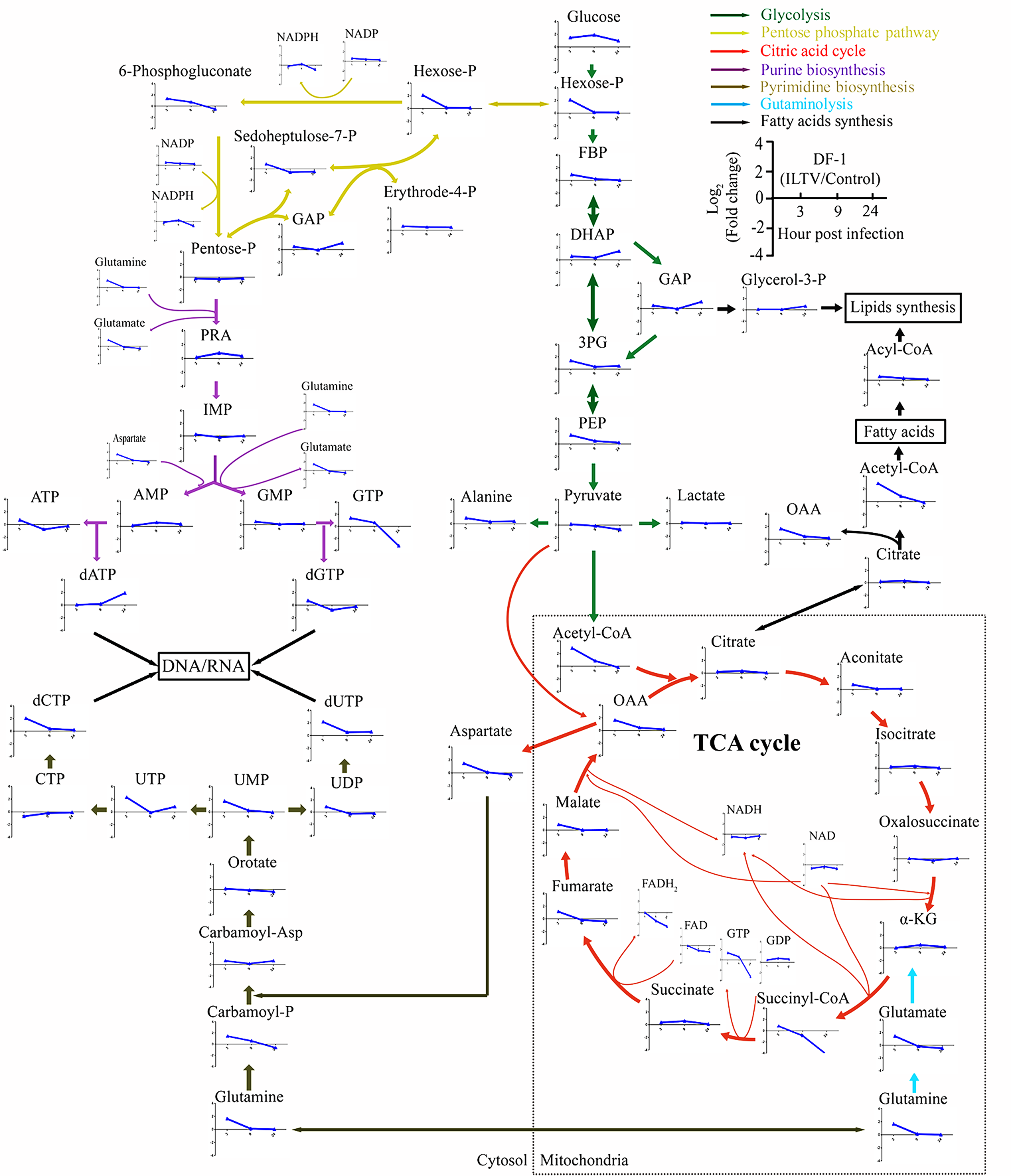

Supplement: S1 Fig — DF-1 cells mock-infected or virally infected at a MOI of 1 with ILTV were harvested at 3, 9, and 24 hpi and processed for LC-MS. Average fold changes in the metabolite levels (relative to the mock-infected samples) of four independent biological experiments are plotted on a log2 axis (n = 4). (Hexose-P: glucose-6-phosphate and its isomers; FBP: fructose-1,6-bisphosphate; DHAP: dihydroxy acetone-phosphate; 3PG: 3-phosphoglycerate; GAP: glyceraldehyde-3-phosphate; PEP: phosphoenolpyruvate; α-KG: α-ketoglutarate; OAA: oxaloacetate; pentose-P: pentose-phosphate; PRA: 5-phosphoribosylamine; PRPP: 5-phosphoribosyl pyrophosphate.). (TIF) [file ppat.1008815.s001.tif]

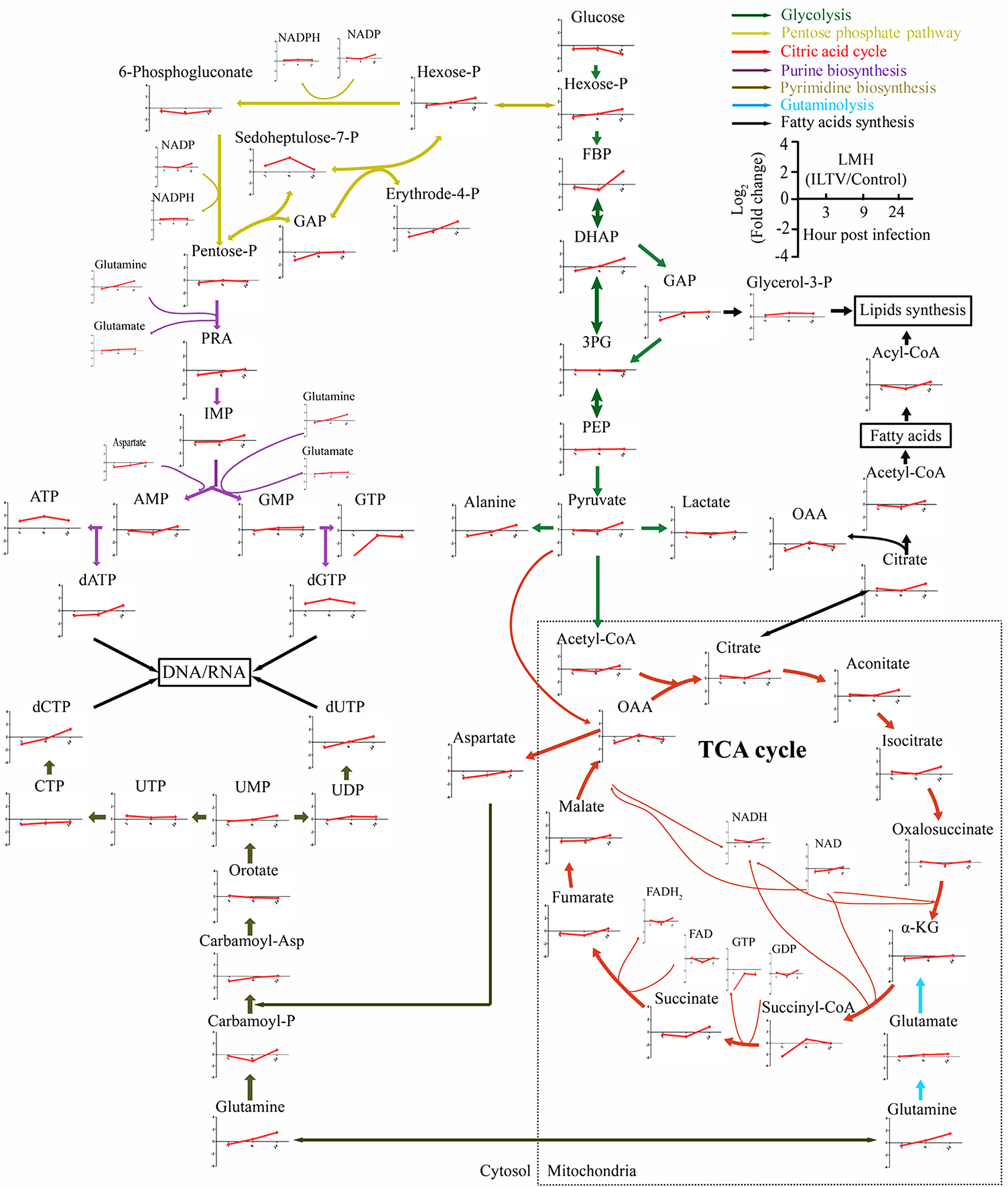

Supplement: S2 Fig — LMH cells mock-infected or virally infected at a MOI of 1 with ILTV were harvested at 3, 9, and 24 hpi and processed for LC-MS. Plots of individual metabolite abundance during ILTV infection are the same as presented in S1 Fig. (TIF) [file ppat.1008815.s002.tif]

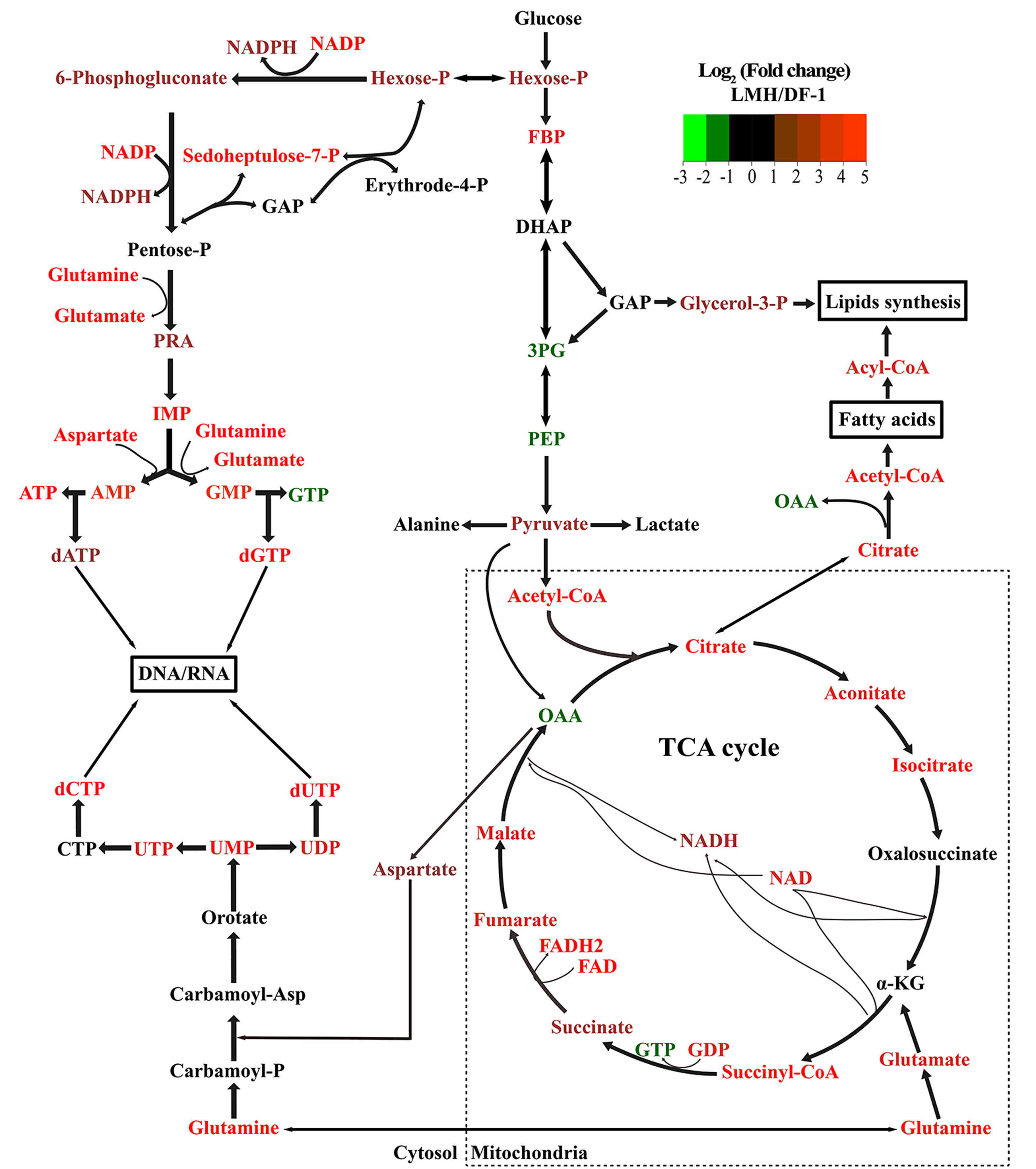

Supplement: S3 Fig — DF-1 cells and LMH cells without ILTV infection were harvested and processed for LC-MS. Average fold changes in the metabolite levels (LMH relative to the DF-1) of four independent biological experiments are log2 transformed and presented as the font color of each metabolite according to the red-green color scale of the scheme (green, the level of indicated metabolite in LMH cells is lower than that in DF-1 cells; red, the level of indicated metabolite in LMH cells is higher than that in DF-1 cells; black, no difference between two cell lines). The abbreviations are the same as presented in S1 Fig. (TIF) [file ppat.1008815.s003.tif]

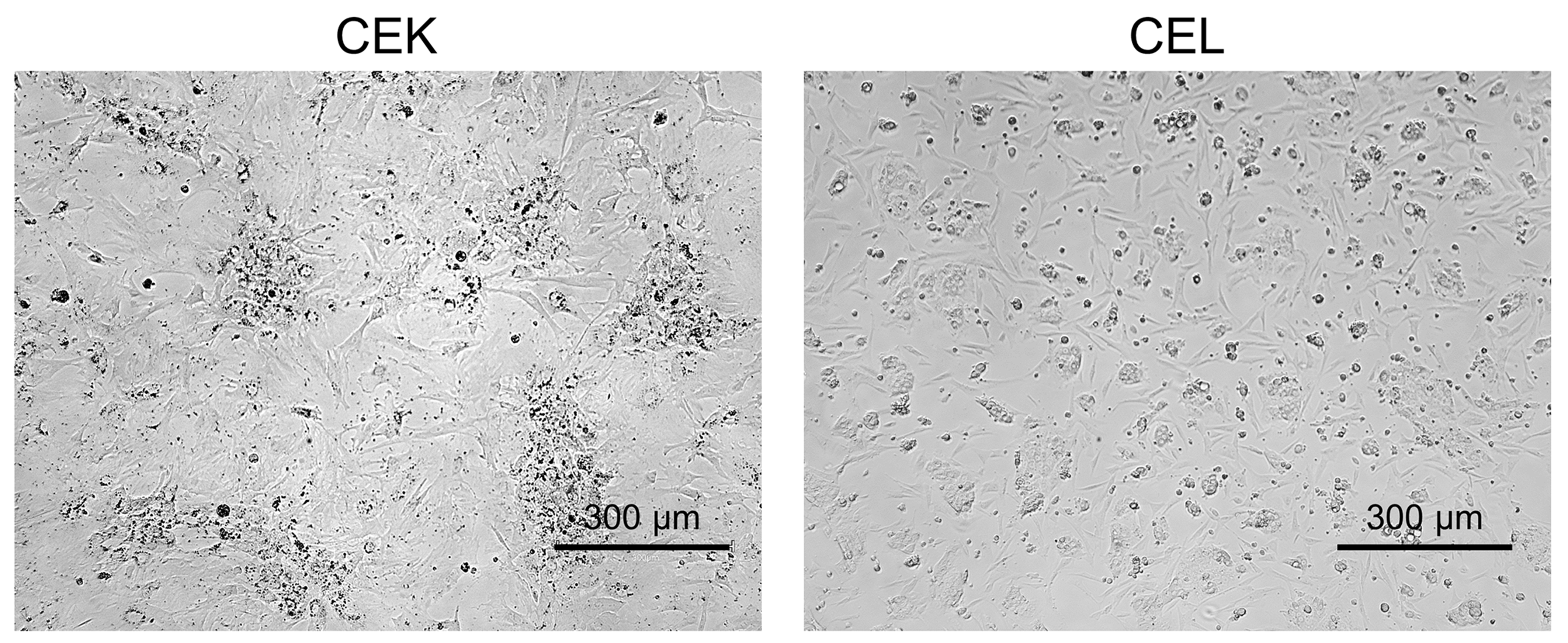

Supplement: S4 Fig — Representative images for primary CEK and primary CEL were obtained by inverted microscopy. The scale bar indicates 300 nm. (TIF) [file ppat.1008815.s004.tif]

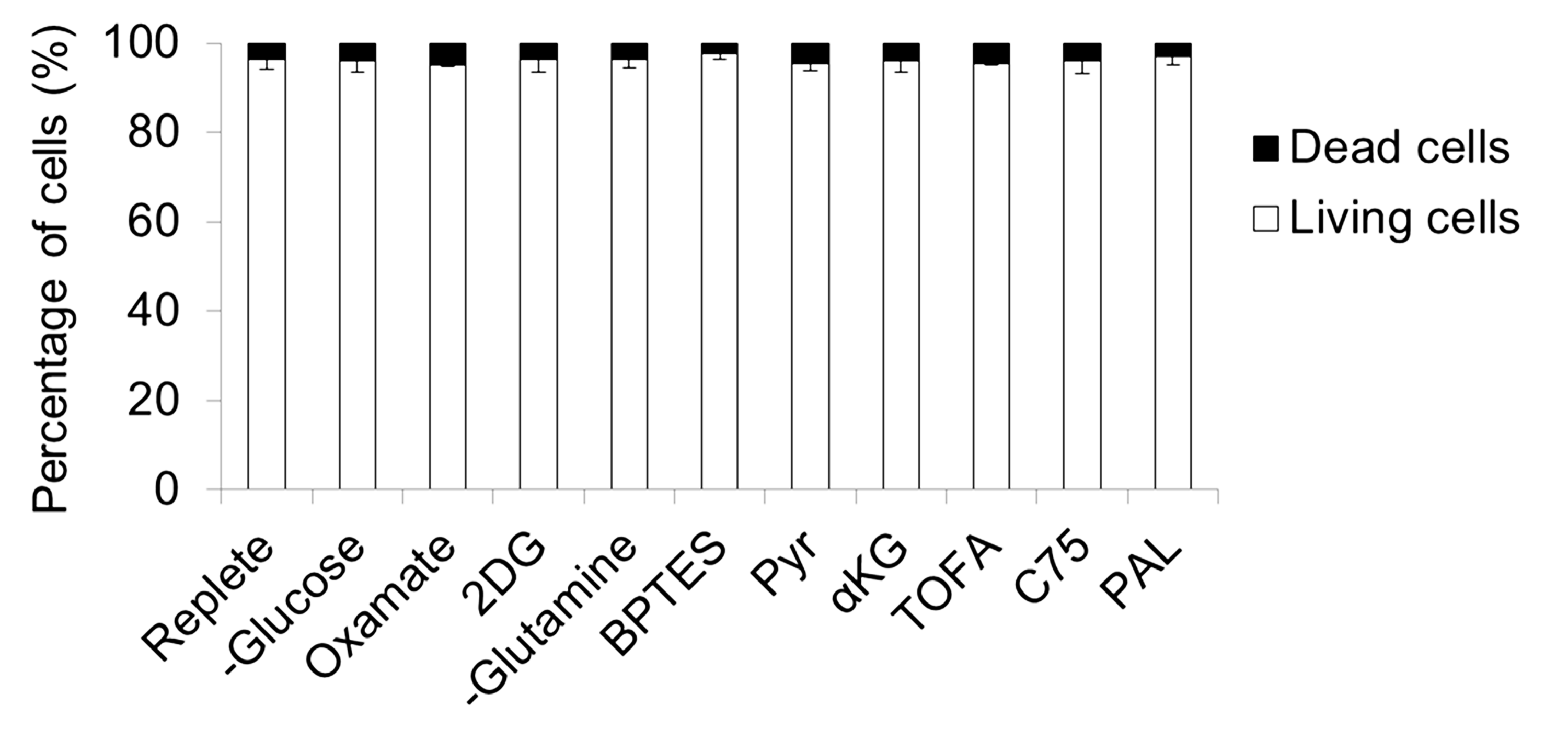

Supplement: S5 Fig — The viability of cells was detected by trypan blue staining according to the manufacturer’s instructions. The results are presented as the mean ± SD, n = 3. Asterisks indicate statistical difference (p < 0.05). (TIF) [file ppat.1008815.s005.tif]
