# Supplementary material for: Global exploration of the metabolic requirements of gallid alphaherpesvirus 1
Source: PLoS Pathog. 2020 Aug 24;16(8):e1008815. doi: 10.1371/journal.ppat.1008815 (PMC7470321; doi:10.1371/journal.ppat.1008815)
Supplement: S3 Table — (DOCX) [file ppat.1008815.s008.docx]

| **S3 Table. List of RT-qPCR primers** | | |
| --- | --- | --- |
| **Genes** |  | **Sequence (5' to 3')** |
| ICP4 | F | AATGCCATACCCAAACCAACT |
|  | R | AATGACAGCCCACCTAATCCC |
| ICP27 | F | GGGCAGTGGATGATGATGATT |
|  | R | GATCCGAAGACAAAAATGCTGG |
| UL48 | F | TGAGGATGATGCGACTGAC |
|  | R | GCGAGAAACGATGCTGAAC |
| gI | F | CACTTTACAGCCGACAAAAACAG |
|  | R | CCAGGAACTTAGCGAGGAG |
| gG | F | GCAACCGCACCACGATTGAGG |
|  | R | TCCACTGCCCGTTTCGCTATCC |
| gC | F | AAATGCTACGACCTGAAACT |
|  | R | CTCGGGCTCATCCAAAACA |
| HK1 | F | AGAAATGAAGAATGGCCTCT |
|  | R | TTCCATTTGTACCGTCTGCTT |
| PKM | F | TTTGCCTCTGACCCGATCACCT |
|  | R | TGTAGTCCACCCACAGCACATT |
| DCN | F | CTTGGCTAAATTGGGTCTCAG |
|  | R | ACTAGGTACTCTGACAAGTTCGT |
| AQP1 | F | AATCATTGGATCTTCTGGGTTGG |
|  | R | TTCACGCGATCAGTCAGG |
| CD99 | F | TGATAACCCCAGGCCAGCTAC |
|  | R | TCACCATCTCCGCCCTTATTT |
| DBNDD2 | F | GCGGCAGAAATTCTTTGAGGAGG |
|  | R | ACAGGCCAGAAATACATCCACA |
| SH3BGRL3 | F | AGAGCGAAGTGACCAGAATCC |
|  | R | AGAGCTCATAATCCCCGCAGT |
| H3-IX | F | CGGAAAAGCGCTCCCTCTAC |
|  | R | CCGCGCTCTGGAACCTCAA |
| GPI | F | CGTTCCAATACCCCAATACT |
|  | R | TCCAGTGTAGCCTTTCCATTC |
| DSTN | F | AAGATGATCTATGCAAGCTCC |
|  | R | CTCCTAGCTTCTCAGCAATACA |
| KARS | F | AATTGGGCTTCCTTGAGATTG |
|  | R | ACTGACGTCCAATTTCATACACT |
| OSTC | F | ATGCACCAAATATCCCCAAGC |
|  | R | GTAGCCCGGTAATTTCATCCTC |
| NPM1 | F | ACTGGACTACGAAGGCAACCCAA |
|  | R | CATAAACAGGCCCCGAACCAC |
| CHCHD2 | F | GCTGCTCCTGCCCCGAAAC |
|  | R | GCCTCAGCGCTGCTTCCTCC |
| RPS20 | F | GATCAGAGGTGCTAAGGAGAAAA |
|  | R | AGTCAATGAGTCGCTTATGG |
| MT-ND2 | F | ATCAAATATTTCCTCACCCAATC |
|  | R | AGGAGCCTTGGAGTACTTCTG |
| PDGF-A | F | AGGATACGATGATGTTTCAGAGAC |
|  | R | GATGGCCTCCTCAATGCT |
| ALDOB | F | TCCAACAGCAAATAAGTCACC |
|  | R | TCCTGAAGCCACAATCCT |
| PPDPF | F | TATCACCGAAGGCGTCTGAG |
|  | R | GGGTCAGATTTGGGCAGAC |
| PHGDH | F | AGCCAAAGCATCGGAGACAA |
|  | R | TGCATTGGAGCTGTGGTAGG |
| LTF | F | ACGATATGACGATGAAAGCCA |
|  | R | TTTCTTGCCCTTCAGATTGTTCC |
| VTN | F | TCTGATGAGACCAGGAACCC |
|  | R | AGTATTTCCCTCGGAAGGCA |
| β-actin | F | GTGGATCAGCAAGCAGGAGT |
|  | R | ATAAAGCCATGCCAATCTCGT |
